# Synthesis and Electrochromic Properties of Triphenylamine-Based Aromatic Poly(amide-imide)s

**DOI:** 10.3390/polym17091152

**Published:** 2025-04-23

**Authors:** Sheng-Huei Hsiao, Zong-De Ni

**Affiliations:** Department of Chemical Engineering and Biotechnology, National Taipei University of Technology, Taipei 10451, Taiwan; ni830314@gmail.com

**Keywords:** triphenylamine, poly(amide-imide)s, electrochemistry, electrochromism, redox-active polymers

## Abstract

Three new amide-preformed triphenylamine-diamine monomers, namely 4,4′-bis(*p*-aminobenzamido)triphenylamine (**4**), 4,4′-bis(*p*-aminobenzamido)-4″-methoxytriphenylamine (**MeO-4**), and 4,4′-bis(*p*-aminobenzamido)-4″-*tert*-butyltriphenylamine (***t*-Bu-4**), were synthesized and subsequently used to produce three series of electroactive aromatic poly(amide-imide)s (PAIs) via two-step polycondensation reactions with commercially available tetracarboxylic dianhydrides. Strong and flexible PAI films could be obtained by solution casting of the poly(amic acid) films followed by thermal imidization or direct solution casting from the organosoluble PAI samples. The PAIs had high glass-transition temperatures of 296–355 °C and showed no significant decomposition below 500 °C. The PAIs based on diamines **MeO-4** and ***t*-Bu-4** showed high electrochemical redox stability and strong color changes upon oxidation. For the PAIs derived from diamine **4**, the TPA radical cation formed in situ during the electro-oxidative process could dimerize to a tetraphenylbenzidine structure, resulting in an additional oxidation state and color change. These PAIs exhibited increased solubility, lowered oxidation potentials, and enhanced redox stability compared to their polyimide analogs.

## 1. Introduction

Electrochromic materials can undergo a reversible optical change in absorption or transmittance upon electrochemical oxidation or reduction. The main kinds of electrochromic materials include metal oxides (such as WO_3_), metal hexacyanoferrates, organic small molecules (such as viologens), and organic polymers (e.g., polyanilines, polythiophenes, and polypyrroles) [1,2]. Among these, organic polymers have several advantages, such as high coloration efficiency, fast response time, compatibility with fabrication into flexible devices, and ease of color tuning [3,4,5,6]. This technology can be applied to smart windows, antiglare rearview mirrors, displays, eyewear, energy storage, and camouflage devices [7,8,9,10]. Electronically dimmable windows developed by Gentex were installed on the Boeing 787 Dreamliner.

Triphenylamine (TPA) derivatives are well known for photoactive and electroactive properties and thus have found optoelectronic applications as photoconductors, hole-transporters, and light-emitters [11,12,13]. TPAs can be easily oxidized to form stable radical cations, and the oxidation process is always associated with a noticeable change in color. In the early 1990s, the synthesis and characterization of polyimides and polyamides containing TPA units were first reported by Imai and colleagues [14,15]. Since 2005, Liou et al. and our research team have reported findings related to the interesting electrochromic properties of high-performance polymers, such as aromatic polyamides and polyimides, carrying the TPA unit as an electrochromic functional moiety [16,17,18]. Many TPA-based electrochromic polymers have since been reported in the literature [19,20,21,22,23,24,25,26]. Yen and Liou have published some comprehensive review papers on TPA-based electrochromic polymers [27,28,29]. The introduction of bulky groups such as a *tert*-butyl group [30,31,32] or electron-donating substituents such as methoxy groups [33,34,35,36] onto the active sites of triarylamine units reduces their oxidation potential and allows them to obtain more stable radical cations, preventing dimerization reactions at these positions.

It has been demonstrated that TPA-based polyimides generally exhibit poor electrochemical and electrochromic stability compared with their polyamide analogs because of the strongly electron-withdrawing imide group, which increases the oxidation potential of the TPA unit and destabilizes the resultant amino radical cation upon oxidation. Incorporating a spacer between the TPA core and the imide ring may improve the electrochemical and electrochromic stability of this kind of electroactive polymer. Thus, three new amide-preformed triphenylamine-diamine monomers, namely 4,4′-bis(*p*-aminobenzamido)triphenylamine (**4**), 4,4′-bis(*p*-aminobenzamido)-4″-methoxytriphenylamine (**MeO-4**), and 4,4′-bis(*p*-aminobenzamido)-4″-*tert*-butyltriphenylamine (***t*-Bu-4**), were synthesized, and a series of poly(amide-imide)s (PAIs) with main-chain TPA and benzamide moieties were prepared from these diamide-preformed diamine monomers with the corresponding tetracarboxylic dianhydrides. As a result of the incorporation of the benzamide spacer between the TPA and imide units, the resulting PAIs are expected to exhibit enhanced electrochemical and electrochromic properties.

## 2. Materials and Methods

### 2.1. Synthesis of 4,4′-Bis(p-nitrobenzamido)triphenylamine (3), 4,4′-Bis(p-nitrobenzamido)-4″-methoxytriphenylamine (MeO-3), and 4,4′-Bis(p-nitrobenzamido)-4″-tert-butyltriphenylamine (t-Bu-3)

In a 250 mL round-bottom flask equipped with a stirring bar, 2.75 g (0.01 mol) of 4,4′-diaminotriphenylamine and 3 mL pyridine were dissolved in 30 mL DMF. A solution of 4.53 g (0.024 mol) of *p*-nitrobenzoyl chloride in 20 mL DMF was added. The reaction mixture was stirred for 3 h and then poured into 600 mL of mixture of methanol and water (2:1). The precipitated solid was collected by filtration, then recrystallized from ethanol and dried to yield 4.36 g (76%) of diamide-dinitro compound **3** as dark red crystals with a melting point of 237~239 °C (by DSC). IR (KBr): 3282 cm^−1^ (amide N–H stretch), 1651 cm^−1^ (amide C=O stretch), 1595, 1350 cm^−1^ (nitro –NO_2_ stretch). ^1^H NMR (600 MHz, DMSO-*d*_6_, δ, ppm): 7.00 (m, 3H, H_g_ + H_e_), 7.05 (d, *J* = 8.9 Hz, 4H, H_d_), 7.30 (t, *J* = 7.8 Hz, 2H, H_f_), 7.73 (d, *J* = 8.9 Hz, 4H, H_c_), 8.18 (d, *J* = 8.9 Hz, 4H, H_b_), 8.35 (d, *J* = 8.9 Hz, 4H, H_a_), 10.55 (s, 2H, amide N–H). ^13^C NMR (150 MHz, DMSO-*d*_6_, δ, ppm): 163.57 (amide carbon), 149.10 (C1), 147.35 (C9), 143.36 (C8), 140.61 (C5), 133.99 (C4), 129.43 (C11), 129.12 (C3), 124.22 (C7), 123.53 (C2), 122.65 (C10), 122.27 (C12), 121.50 (C6). Anal. calcd. for C_32_H_23_N_5_O_6_ (573.57): C, 67.01%; H, 4.04%; N, 12.21%. Found: C, 66.89%; H, 4.21%; N, 12.10%.


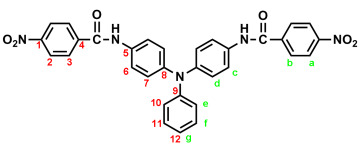


By a similar procedure, diamide-dinitro compound **MeO-3** was synthesized from the condensation of 3.05 g (0.01 mol) of 4,4′-diamino-4″-methoxytriphenylamine (**MeO-2**) and 4.53 g (0.024 mol) of *p*-nitrobenzoyl chloride as an orange powder (4.23 g; 70% yield) with a melting point of 219~221 °C. IR (KBr): 3286 cm^−1^ (amide N–H stretch), 1651 cm^−1^ (amide C=O stretch), 1600, 1350 cm^−1^ (nitro –NO_2_ stretch). ^1^H NMR (600 MHz, DMSO-*d*_6_, δ, ppm): 3.75 (s, 3H, methoxy), 6.94 (d, *J* = 8.9 Hz, 2H, H_e_), 6.96 (d, *J* = 8.9 Hz, 4H, H_d_), 7.03 (d, *J* = 8.9 Hz, 2H, H_f_), 7.68 (d, *J* = 8.9 Hz, 4H, H_c_), 8.18 (d, *J* = 8.8 Hz, 4H, H_b_), 8.36 (d, *J* = 8.8 Hz, 4H, H_a_), 10.05 (s, 2H, amide N–H). ^13^C NMR (150 MHz, DMSO-*d*_6_, δ, ppm): 163.44 (amide carbon), 155.71 (C12), 149.06 (C1), 143.96 (C8), 140.64 (C5), 140.09 (C9), 133.05 (C4), 129.08 (C3), 126.45 (C11), 123.51 (C2), 122.60 (C7), 121.73 (C6), 115.01 (C10), 55.24 (–OCH_3_). Anal. calcd. for C_33_H_25_N_5_O_7_ (603.59): C, 65.67%; H, 4.17%; N, 11.60%. Found: C, 65.21%; H, 4.23%; N, 11.30%.


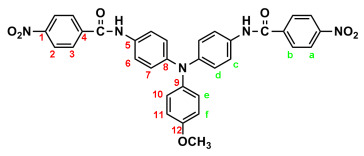


Similarly, diamide-dinitro compound **t-Bu-3** was synthesized from the condensation of 3.31 g (0.01 mol) of 4,4′-diamino-4″-tert-butyltriphenylamine (**t-Bu-2**) and 4.53 g (0.024 mol) of p-nitrobenzoyl chloride as dark-red crystals (5.35 g; 85% yield) with a melting point of 223~224 °C. IR (KBr): 3300 cm^−1^ (amide –N–H stretch), 1655 cm^−1^ (amide C=O stretch), 1596, 1350 cm^−1^ (nitro –NO_2_ stretch). ^1^H NMR (600 MHz, DMSO-d_6_, δ, ppm): 1.27 (s, 9H, H_g_), 6.94 (d, J = 6.8 Hz, 2H, H_e_), 7.02 (d, J = 8.9 Hz, 4H, H_d_), 7.32 (d, J = 6.8 Hz, 2H, H_f_), 7.71 (d, J = 8.9 Hz, 4H, H_c_), 8.18 (d, J = 7.8 Hz, 4H, H_b_), 8.36 (d, J = 7.9 Hz, 4H, H_a_), 10.53 (s, 2H, amide N–H). Anal. calcd. for C_36_H_31_N_5_O_6_ (629.67): C, 68.67%; H, 4.96%; N, 11.12%. Found: C, 68.20%; H, 5.03%; N, 11.01%.


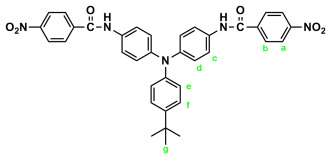


### 2.2. Synthesis of 4,4′-Bis(p-aminobenzamido)triphenylamine (4), 4,4′-Bis(p-aminobenzamido)-4″methoxytriphenylamine (MeO-4), and 4,4′-Bis(p-aminobenzamido)-4″-tert-butytriphenylamine (t-Bu-4)

In a 100 mL three-neck round-bottom flask equipped with a stirring bar, 1.72 g (0.003 mol) of diamide-dinitro compound **3** and 0.15 g of 10% Pd/C were dispersed in 50 mL of ethanol. The equivalent amount of hydrazine was added to the mixture, and the solution was stirred at a reflux temperature for about 6 h. The solution was then filtered to remove Pd/C, and the filtrate was concentrated in a rotatory evaporator and cooled to precipitate the product, which was collected by filtration and dried in vacuum to yield 1.08 g (70%) of diamide–diamino compound **4**, which had a a melting point of 261~264 °C. IR (KBr): 3350, 3300 cm^−1^ (amide and amino N–H stretch), 1630 cm^−1^ (amide C=O stretch). ^1^H NMR (600 MHz, DMSO-*d*_6_, δ, ppm): 5.72 (s, 4H, –NH_2_), 6.60 (d, *J* = 8.7 Hz, 4H, H_a_), 6.92–6.94 (two overlapped doublets, 3H, H_g_ + H_e_), 6.98 (d, *J* = 8.9 Hz, 4H, H_d_), 7.24 (t, *J* = 7.8 Hz, 2H, H_f_), 7.68 (d, *J* = 8.9 Hz, 4H, H_c_), 7.70 (d, *J* = 8.7 Hz, 4H, H_b_), 9.74 (s, 2H, amide N–H). ^13^C NMR (150 MHz, DMSO-*d*_6_, δ, ppm): 165.04 (amide carbon), 152.03 (C1), 147.76 (C9), 142.29 (C8), 135.23 (C5), 129.23 (C3,C11), 124.41 (C7), 121.68 (C10), 121.43 (C12), 121.38 (C6), 121.12 (C4), 112.51 (C2). Anal. calcd. for C_32_H_27_N_5_O_2_ (513.59): C, 74.84%; H, 5.29%; N, 13.64%. Found: C, 74.09%; H, 5.40%; N, 13.23%.


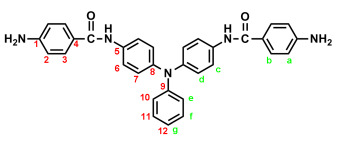


By a similar procedure, diamide–diamines **MeO-4** and ***t*-Bu-4** were synthesized by hydrazine Pd/C-catalyzed reduction of diamide-dinitro compounds **MeO-3** and ***t*-Bu-3**, respectively. The spectroscopic and microanalysis data are shown below.

IR for **MeO-4** (KBr): 3500~3300 cm^−1^ (amine and amide N–H stretch), 1623 cm^−1^ (amide C=O stretch). ^1^H NMR for **MeO-4** (600 MHz, DMSO-*d_6_*, δ, ppm): 3.75 (s, 3H, methoxy), 5.70 (s, 4H, –NH_2_), 6.59 (d, *J* = 7.8 Hz, 4H, H_a_), 6.90 (two overlapped doublets, 6H, H_d_ + H_e_), 6.98 (d, *J* = 9.0 Hz, 4H, H_f_), 7.62 (d, *J* = 6.9 Hz, 4H, H_c_), 7.70 (d, *J* = 7.8 Hz, 4H, H_b_), 9.69 (s, 2H, amide N–H). ^13^C NMR for **MeO-4** (150 MHz, DMSO-*d*_6_, δ, ppm): 164.97 (amide carbon), 155.26 (C12), 151.97 (C1), 143.08 (C8), 140.56 (C9), 134.20 (C5), 129.20 (C3), 125.86 (C11), 122.69 (C7), 121.37 (C6), 121.21 (C4), 114.87 (C10), 112.53 (C2), 55.21 (–OCH_3_). Anal. calcd. for C_33_H_29_N_5_O_3_ (543.62): C, 72.91%; H, 5.37%; N, 12.88%. Found: C, 72.19%; H, 5.50%; N, 12.03%.


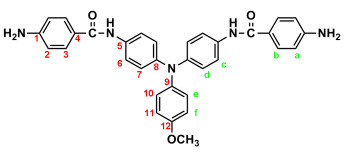


IR for ***t*-Bu-4** (KBr): 3500~3300 cm^−1^ (amine and amide N–H stretch), 1623 cm^−1^ (amide C=O stretch). ^1^H NMR for ***t*-Bu-4** (600 MHz, DMSO-*d_6_*, δ, ppm): 1.27 (s, 9H, H_g_), 5.72 (s, 4H, –NH_2_), 6.60 (d, *J* = 8.6 Hz, 4H, H_a_), 6.89 (d, *J* = 8.7 Hz, 2H, H_e_), 6.95 (d, *J* = 8.9 Hz, 4H, H_d_), 7.28 (d, *J* = 8.7 Hz, 2H, H_f_), 7.66 (d, *J* = 8.9 Hz, 4H, H_c_), 7.70 (d, *J* = 8.6 Hz, 4H, H_b_), 9.72 (s, 2H, amide N–H). Anal. calcd. for C_36_H_35_N_5_O_2_ (569.70): C, 75.89%; H, 6.19%; N, 12.29%. Found: C, 75.10%; H, 6.30%; N, 12.11%.


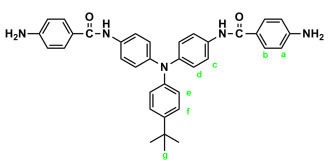


### 2.3. Synthesis of Poly(amide-imide)s

The PAIs were prepared from various tetracarboxylic dianhydrides (PMDA, BPDA, BTDA, ODPA, DSDA, and 6FDA) with amide-performed diamino compounds **4**, **MeO-4** and ***t*-Bu-4**, respectively, by a conventional two-step method via thermal or chemical imidization reaction. The synthesis of PAI **6f** is described as an example. Into a solution of amide-performed diamino compound **4** (0.5362 g; 1.04 mmol) in 9.5 mL anhydrous DMAc in a 50 mL round-bottom flask, 0.4638 g (1.04 mmol) of 6FDA (**5f**) was added in one portion. Thus, the solid content of the solution was approximately 10 wt%. The mixture was stirred at room temperature for 6 h to yield a viscous poly(amide-amic acid) (PAA) solution with an inherent viscosity of 0.68 dL/g, as measured in DMAc at concentration of 0.50 g/dL at 30 °C. The poly(amide-amic acid) film was obtained by casting from the reaction polymer solution onto a glass Petri dish and drying at 90 °C overnight. The poly(amide-amic acid) in the form of a solid film was converted to the PAI film by successive heating steps at 150 °C for 30 min, 200 °C for 30 min, and 250 °C for 1 h. For chemical imidization, 2 mL of acetic anhydride and 1mL of pyridine were added to a PAA solution obtained by a process similar to that described above, and the mixture was heated at 100 °C for 1 h to effect a complete imidization. The homogenous polymer solution was poured slowly into an excess of methanol, giving rise to a precipitate that was collected by filtration, washed thoroughly with hot water and methanol, and dried. The inherent viscosity of the resulting PAI **6f** was 0.64 dL/g, as measured in DMAc at concentration of 0.50 g/dL at 30 °C. A polymer solution was made by the dissolution of about 0.5 g of the PAI sample in 4 mL of hot DMAc. The homogeneous solution was poured into a glass Petri dish, which was placed in a 90 °C oven overnight for slow release of solvent, and then the film was stripped off from the glass substrate and further dried in vacuum at 160 °C for 6 h. The IR spectrum of **6f** (film) exhibited characteristic imide and amide absorption bands at 1786 cm^−1^ (asymmetrical imide C=O stretch), 1725 cm^−1^ (symmetrical imide stretch), and 1650 cm^−1^ (amide C=O stretch).

## 3. Results and Discussion

### 3.1. Monomer Synthesis

**Scheme 1:**
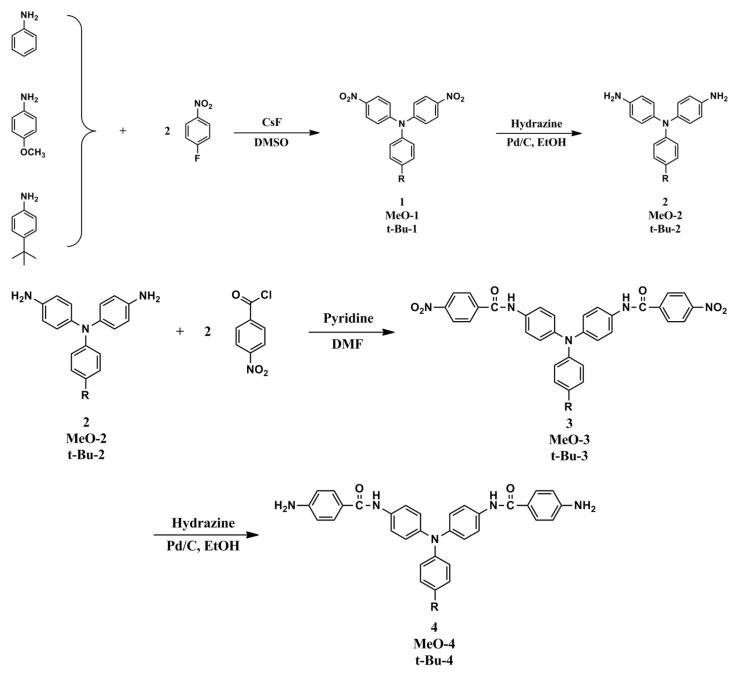
Synthetic routes to TPA-based diamide–diamine monomers **4**, **MeO-4** and ***t*-Bu-4**.

The target TPA-based diamide–diamine monomers **4**, **MeO-4** and ***t*-Bu-4** were synthesized by a four-step reaction sequence, as shown in Scheme 1. According to a well-known synthetic method [14], CsF-assisted *N*,*N*-diarylation of aniline, *p*-anisidine and *p*-*tert*-butylaniline, respectively, with two equivalent amount of *p*-fluoronitrobenzene in DMSO gave the TPA-dinitro compounds 4,4-dinitrotriphenylamine (**1**), 4-methoxy-4′,4″-dinitrotriphenylamine (**MeO-1**), 4-*tert*-butyl-4′,4″-dinitrotriphenylamine (***t*-Bu-1**), which were subsequently converted to the TPA-diamines 4,4′-diaminotriphenylamine (**2**), 4,4′-diamino-4″-methoxytriphenylamine (**MeO-2**), and 4,4′-diamino-4″-*tert*-butyl-triphenylamine (***t*-Bu-2**) by hydrazine Pd/C-catalyzed reduction. The targeted diamide–diamine monomers 4,4′-bis(*p*-aminobenzamido)triphenylamine (**4**), 4-bis(*p*-aminobenzamido)-4″-methoxytriphenylamine (**MeO-4),** and 4,4-bis(*p*-aminobenzamido)-4″-*tert*-butyltriphenylamine (***t*-Bu-4**) were prepared by condensation of **2**, **MeO-2** and ***t*-Bu-2** with two equivalent amount of *p*-nitrobenzoyl chloride and followed by hydrazine Pd/C-catalyzed reduction of the intermediate diamide-dinitro compounds **3**, **MeO-3** and ***t*-Bu-3**. All the synthesized compounds were characterized by FT-IR and ^1^H NMR spectroscopy. The diamide-dinito and diamide–diamino compounds were also characterized by ^13^C NMR spectroscopy.

Figure S1 in the Supplemental Materials illustrates the FT-IR spectra of compounds **1**–**4**. The nitro groups of **1** show the characteristic absorptions at 1579 cm^−1^ and 1350 cm^−1^ (–NO_2_ asymmetric and symmetric stretching). After reduction, the characteristic absorptions of the nitro group disappeared, and **2** shows the typical –NH_2_ stretching absorption pair at 3421 cm^−1^ and 3341 cm^−1^. Compound **3** shows the characteristic amide absorption at 3282 cm^−1^ (N–H stretching) and 1651 cm^−1^ (amide C=O stretching) and shows the characteristic absorptions at 1595 cm^−1^ and 1350 cm^−1^ (–NO_2_ asymmetric and symmetric stretching). After reduction, the characteristic absorptions of nitro group disappeared, and **4** shows the typical –NH_2_ stretching absorption bands at 3300 and 3350 cm^−1^ (amide and amine N–H stretching) and 1630 cm^−1^ (amide C=O stretching). The IR spectra of the diamide–diamines **MeO-4** and ***t*-Bu-4** and their precursor compounds are also included in the Supplemental Materials (Figure S2).

**Figure 1:**
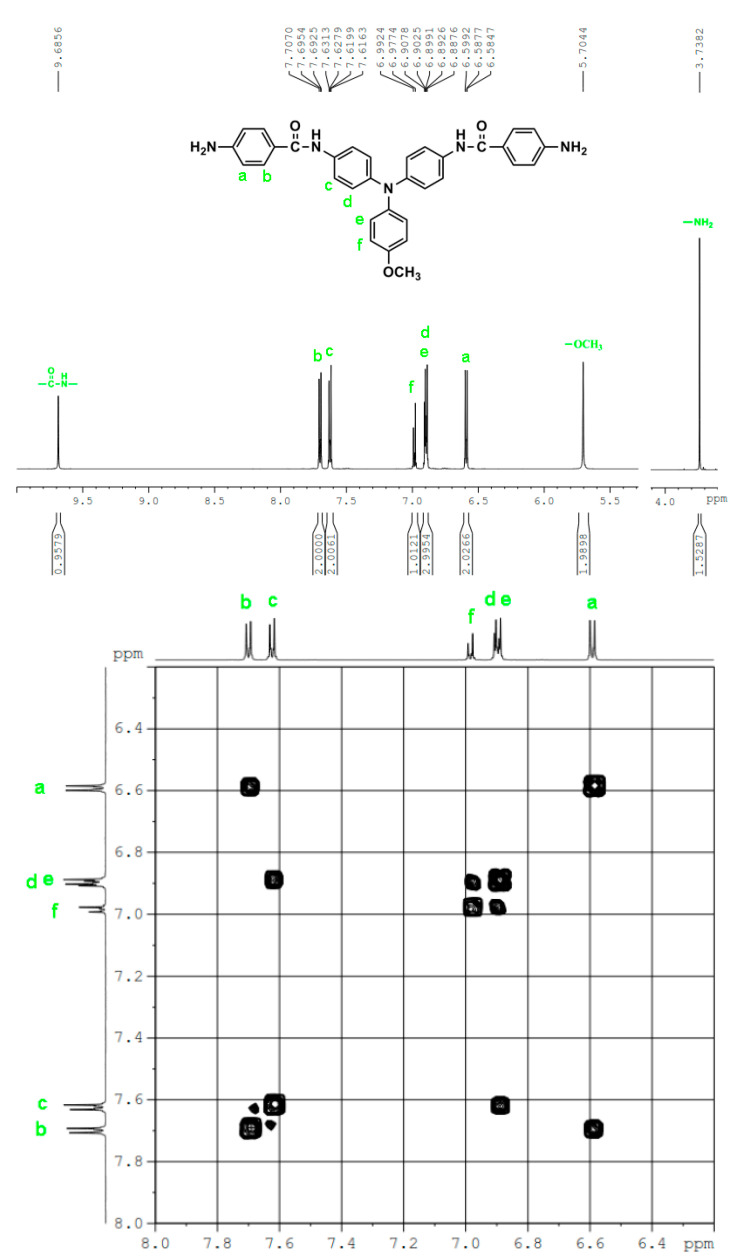
^1^H NMR and H-H COSY of diamide–diamine **MeO-4** in DMSO-*d*_6_.

**Figure 2:**
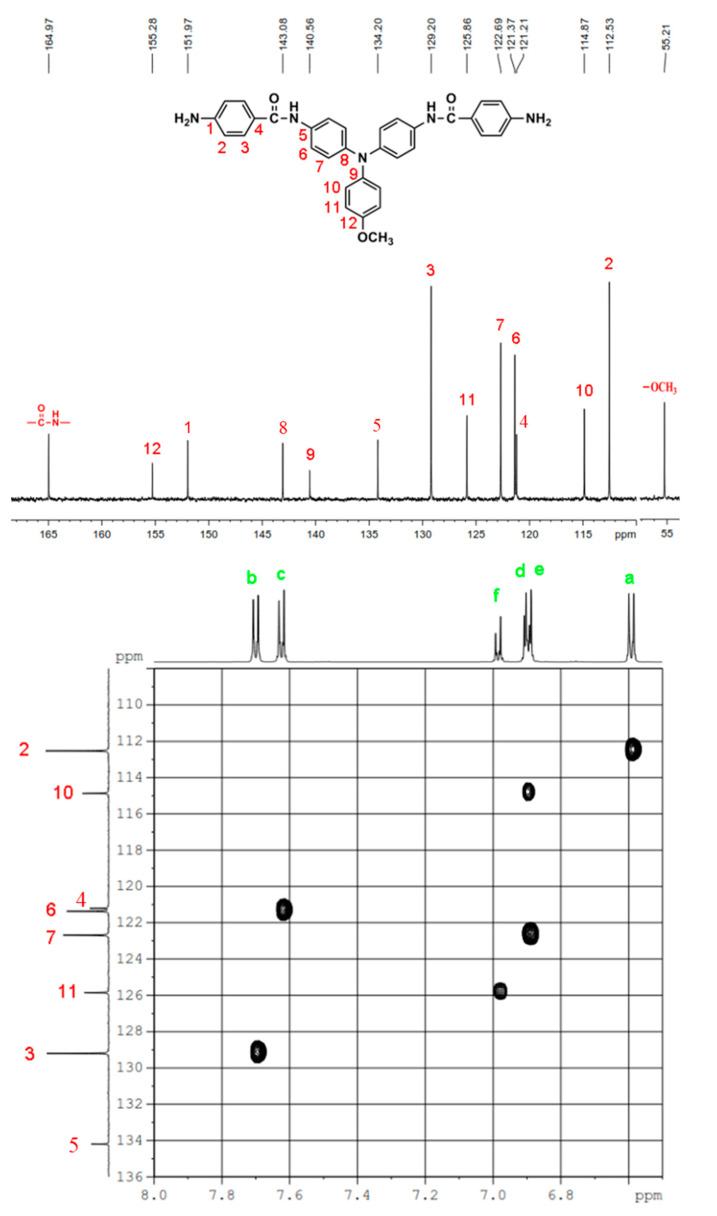
^13^C NMR and C-H HMQC of diamide–diamine **MeO-4** in DMSO-*d*_6_.

The molecular structures of all the TPA-based diamide-dinitro and diamide–diamino compounds were also confirmed by ^1^H and ^13^C NMR spectra. As a typical example, the ^1^H NMR and H-H COSY of the diamide–diamine monomer **MeO-4** in DMSO-*d*_6_ are illustrated in Figure 1, and its ^13^C NMR and C-H HMQC are shown in Figure 2. The assignments of resonance peaks were assisted by 2D NMR spectra, and all the NMR spectra are in good agreement with the molecular structure of **MeO-4**. The NMR spectra of the other synthesized compounds are summarized in the Supplemental Materials Figures S3–S7. Thus, the IR and NMR spectra confirmed that all the compounds reported herein were successfully synthesized.

### 3.2. Synthesis of Poly(amide-imide)s (PAIs)

**Scheme 2:**
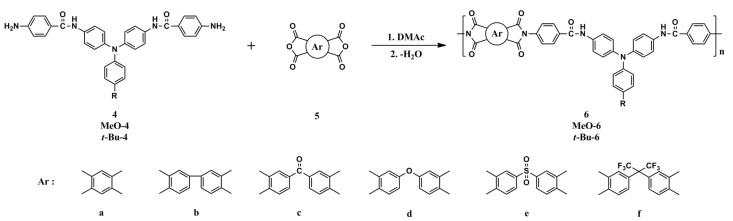
Synthesis of poly(amide-imide)s.

Three series of aromatic PAIs (**6a-f**, **MeO-6a-f** and ***t*-Bu-6a-f**) with TPA units were prepared via a conventional two-step method that involved the reaction of equimolar amounts of the diamines **4**, **MeO-4** and ***t*-Bu-4** with various aromatic dianhydrides (**5a**–**5f**) to form poly(amide-amic acid)s. This step was followed by thermal or chemical cycloehydration (Scheme 2). As shown in Table 1, the poly(amide-amic acid) precursors had inherent viscosities in the range of 0.29–0.91 dL/g. The molecular weights of these poly(amide-amic acid)s were sufficiently high to permit the casting of flexible and tough poly(amide-amic acid) films, which were subsequently converted into tough PAI films by stage-by-stage heating to elevated temperatures. The inherent viscosities of these thermally imidixed PAIs were in the range 0.35–0.88 dL/g, as measured at a concentration of 0.5 dL/g in DMAc at 30 °C. The transformation from poly(amide-amic acid) to a PAI could also be carried out via chemical cyclodehydration by using acetic anhydride and pyridine.

The structures of the PAIs were confirmed by IR and NMR spectroscopy. A typical pair of IR spectra, those of PAI **6f** and its poly(amide-amic acid) precursor, is illustrated in Figure S8. PAI **6f** exhibited characteristic imide-group absorptions around 1786 and 1725 cm^−1^ (typical of imide carbonyl asymmetrical and symmetrical stretch). The proton NMR spectra of PAIs **6f**, **MeO-6f** and ***t*-Bu-6f** in DMSO-*d*_6_ are shown in Figure S9, with the resonance peaks clearly assigned to the repeating units of the polymer backbone. All the aromatic protons in the TPA moiety resonated in the region of δ 7.62–6.91 ppm; the protons of the 6FDA component appeared at 8.22–7.77 ppm; and protons on the benzamide unit appeared at 8.07–7.74 ppm.

### 3.3. Solubility and Thermal Properties

The qualitative solubility properties of the polymers in several organic solvents at a concentration of 10% (*w*/*v*), along with their inherent viscosities, are summarized in Table 1. Most of the PAIs were easily soluble in polar organic solvents such as NMP, DMAc, DMF, and DMSO, whether at room temperature or on heating. Some of them were even soluble in the less-polar solvent *m*-cresol upon heating at 60 °C. Therefore, they could be easily solution-cast into flexible and tough films.

**Figure 3:**
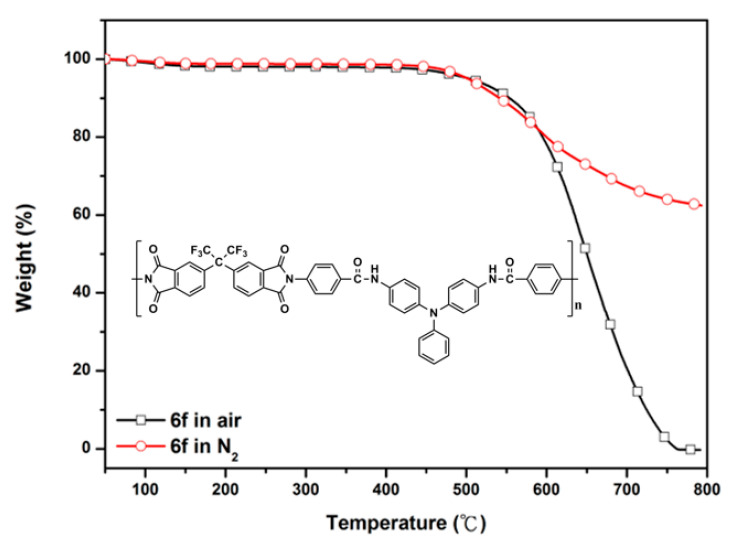
Typical TGA thermograms of PAI **6f** with a heating rate of 20 °C/min in air and nitrogen.

The thermal properties of the polymers were investigated by DSC and TGA. The relevant data are summarized in Table 2. DSC measurements were conducted at a heating rate of 20 °C/min under a nitrogen flow. Quenching from an elevated temperature of about 400 °C to 50 °C yielded predominantly amorphous samples, allowing the glass-transition temperature (*T*_g_) of these PAIs to be easily measured in the second heating cycle of the DSC traces. Typical DSC traces of the **6** series PAIs are shown in Figure S10. The glass-transition temperature (*T*_g_) is defined as the temperature at the midpoint of the baseline shift. These PAIs exhibited moderately high *T*_g_ in the range 286 to 355 °C. The PAIs derived from ODPA (**5d**) exhibited the lowest *T*_g_ values in the series because of the presence of a flexible ether linkage in the dianhydride component. The thermal stability of the polymers was evaluated by TGA in both air and nitrogen atmospheres. The TGA curves of PAI **6f** measured in nitrogen and in air are illustrated in Figure 3. These polymers exhibited reasonable thermal stability without significant weight loss up to 500 °C under a nitrogen or air atmosphere. The decomposition temperatures (*T*_d_) at 5% and 10% weight losses in nitrogen and air atmospheres, taken from the original TGA thermograms, are given in Table 2.

### 3.4. Electrochemical Properties

The electrochemical behavior of the polymer was investigated by cyclic voltammetry (CV) on the cast film on an ITO-coated glass substrate as the working electrode; the experiment was carried out in dry acetonitrile (MeCN) containing 0.1 M of tetrabutylammonium perchlorate (TBAP; Bu_4_NClO_4_) as the supporting electrolyte and saturated Ag/AgCl as the reference electrode under a nitrogen atmosphere. The derived oxidation potentials are summarized in Table 3. As illustrated in Figure S11, all PAIs (**6a-6f**) with non-substituted TPA show reversible oxidation in the initial CV scans, with oxidation peak potentials (*E*_pa_) at about 0.90–0.98 V and onset potentials (*E*_onset_) at 0.70–0.72 V that correspond to the TPA oxidation. In contrast, the **MeO-6** and ***t*-Bu-6** series PAIs with methoxy or *tert*-butyl-substituted TPA (CV diagrams shown in Figures S13 and S15) have lower oxidation peak and onset potentials (*E*_onset_ at 0.56–0.59V and *E*_pa_ at about 0.78–0.88 V of methoxy-substituted polymer; *E*_onset_ at 0.63–0.68 V and *E*_pa_ at about 0.86–0.97 V of *tert*-butyl-substituted polymer) than non-substituted polymers. Because of the electron-donating property of methoxy and *tert*-butyl groups, the nitrogen center of TPA can be oxidized easily. The **MeO-6** series PAIs have the lowest oxidation potentials due to the group’s strong electron-donating effect. During repetitive scans between 0 and 1.2 V, the redox waves of the non-substituted PAIs exhibited slight broadening due to coupling reactions of the TPA units (see Figure S12). The CV-scanned films of the **6** series PAIs were essentially insoluble in sulfuric acid or NMP. On the other hand, the methoxy- or *t*-butyl-substituted polymers displayed very high electrochemical stability. After 50 repeated cycles, their CV curves remained almost identical to those from the initial scans (Figures S14 and S16). The energy levels of the highest occupied molecular orbital (HOMO) and lowest unoccupied molecular orbital (LUMO) of the corresponding polymers were estimated from the *E*_1/2_^Ox^ values [18,30]. The redox potentials and energy levels of all the polymers are summarized in Table 3. Assuming that the HOMO energy level for the ferrocene/ferrocenium (Fc/Fc^+^) standard is 4.80 eV with respect to the zero-vacuum level, the HOMO energy levels for the PAIs were calculated (from *E*_1/2_^Ox^ values) to be in the range 5.07–5.20 eV. The LUMO/HOMO energy gaps estimated from the absorption spectra were then used to obtain the LUMO energy levels.

**Figure 4:**
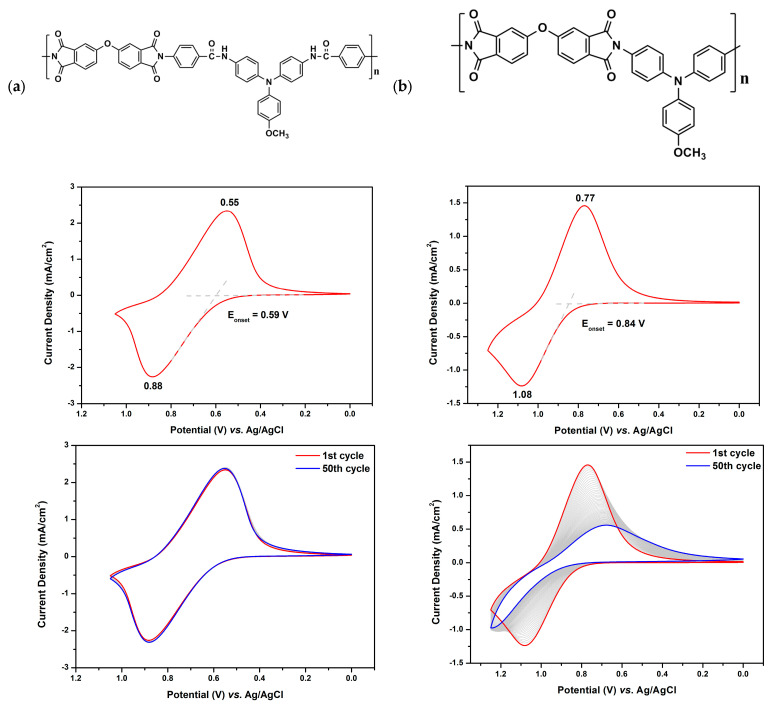
CV diagrams of PAIs (**a**) **MeO-6d** and (**b**) **MeO-6′d** on an ITO-glass slide in MeCN (0.1 M Bu_4_NClO_4_) at a scan rate 50 mV/s. Top: the first CV scans; bottom: after 50 repeated CV scans.

The effect of inserting a benzamide unit on the electrochemical stability of the polymer can be seen in Figure 4. A polymer without benzamide, like **MeO-6′d**, shows a higher oxidation onset potential, at 0.84 V, and anodic peak potential, at 1.08 V. After fifty repeated cycles, the CV diagram of **MeO-6d** shows little change from the initial scan, implying that the compound has very high electrochemical stability. In contrast, **MeO-6′d**, which lacks the benzamide spacer, shows low electrochemical stability on repeated cycling. Therefore, inserting the benzamide spacer to separate the TPA and imide ring enhances the stability of the TPA radical cations formed in situ during the redox processes and thus can improve the electrochemical stability of these PAIs.

### 3.5. Spectroelectrochemical Properties

Spectroelectrochemical measurements were performed on films of polymers drop-coated onto ITO-coated glass slides immersed in an electrolyte solution. The electrode preparations and solution conditions were identical to those used in the CV experiments. During the test, a three-electrode configuration was used for applying potential to the polymer films in a 0.1 M Bu_4_NClO_4_/CH_3_CN electrolyte solution. When the films were electrochemically oxidized, a strong color change was observed.

**Figure 5:**
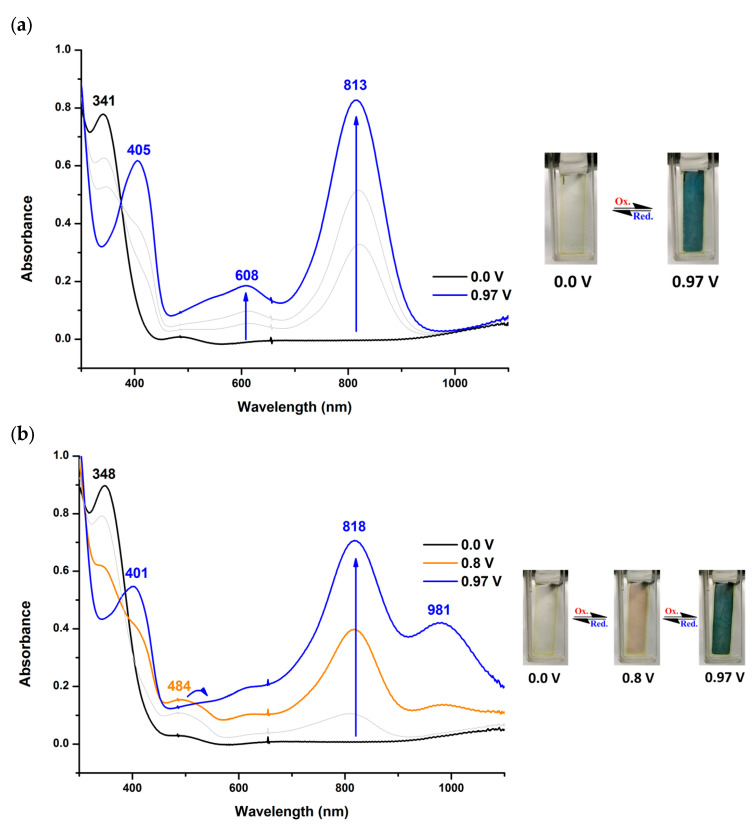
Spectroelectrograms and color changes of PAI **6d** on an ITO-glass slide in 0.1 M Bu_4_NClO_4_MeCN at various applied voltages: (**a**) first cycle and (**b**) after 50 cycles.

**Scheme 3:**
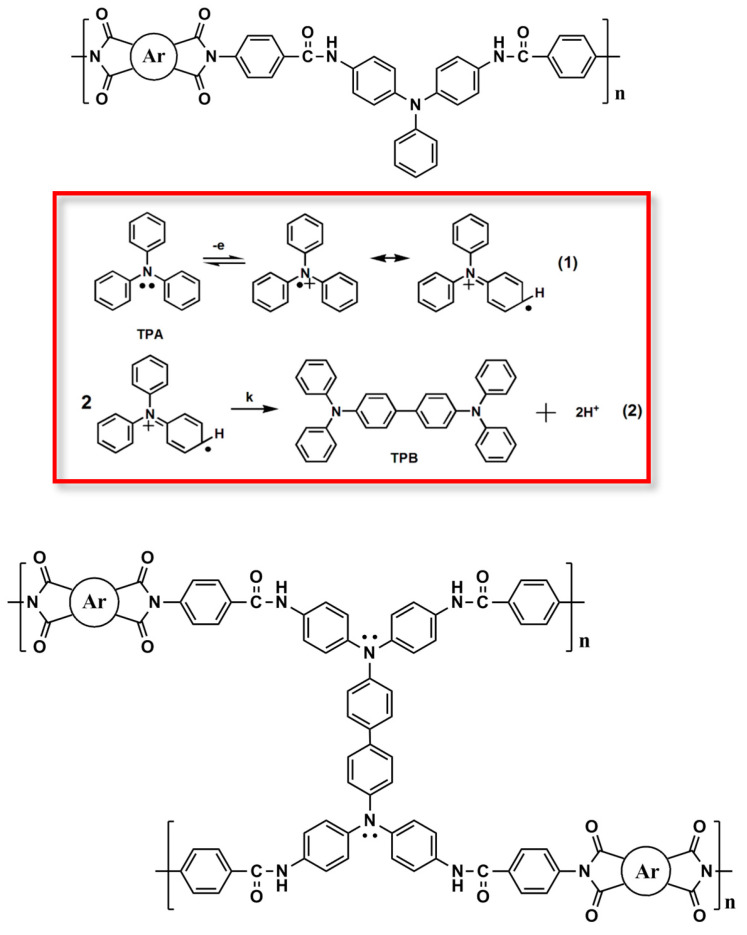
The proposed coupling reaction of TPA units of PAI **6d** during applied voltage cycling between 0 and 1.0 V.

**Figure 6:**
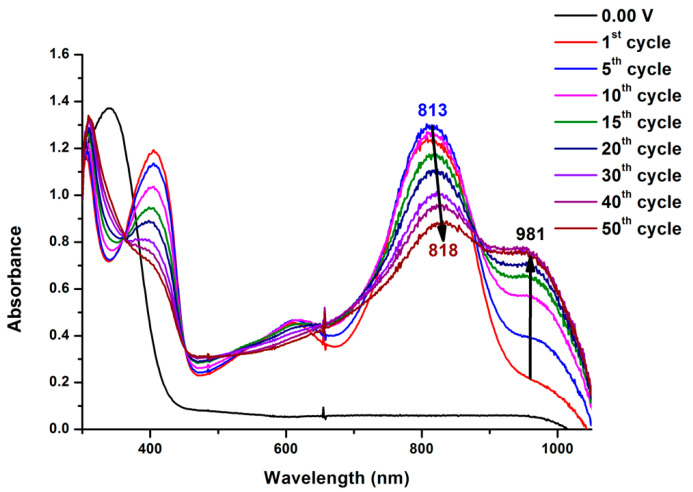
Spectral changes of polymer film **6d** on an ITO-glass slide in 0.1 M Bu_4_NClO_4_/MeCN after various scanning cycles at an applied voltage of 1.0 V.

As shown in Figure 5, the film of PAI **6d** with non-substituted TPA exhibited a strong absorption at a wavelength around 341 nm in the neutral form. Upon oxidation, two main bands appeared at 405 and 813 nm, while the main band around 341 nm that was associated with the neutral state gradually decreased in intensity. The long-wavelength absorption band that appears upon oxidation is characteristic of the cation-radical form. At the same time, the film turned from colorless (L*: 74; a*: 0; b*: 7) to blue-green (L*: 38; a*: −12; b*: 6) with the application of increasing voltages. When this polymer was repeatedly scanned for fifty cycles, we found that when the voltage was set to 0.8 V, a new peak appeared around 484 nm and the film changed color to yellowish orange (L*: 63; a*: 4; b*: 17). When the applied voltage increased, new absorption bands appeared around 818 and 981 nm and the color of the film changed to blue-green. The evolution of the new orange color and the different absorption spectra imply that TPA underwent a coupling reaction to form the tetraphenylbenzidine (TPB) moiety, as shown in Scheme 3. Figure 6 depicts the spectral change of PAI **6d** after various scanning cycles at 1.0 V. As the number of scanning cycles increased, the absorption peak at 981 nm intensified and the peak at 813 nm gradually shifted to 818 nm. The other **6** series PAIs showed spectra and color changes similar to those of **6d**.

**Figure 7:**
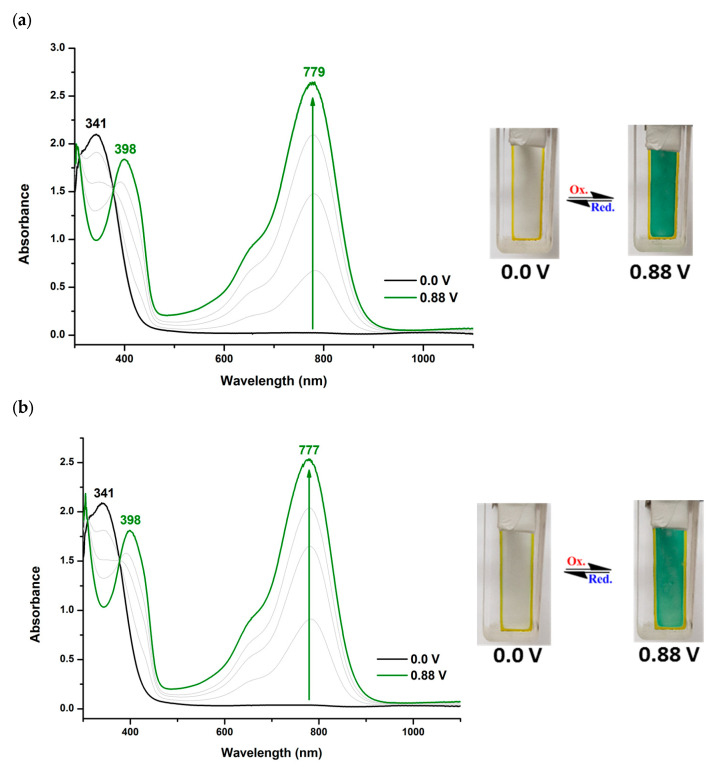
Spectroelectrograms and color changes of PAI **MeO-6d** on an ITO-glass slide in 0.1 M Bu_4_NClO_4_/MeCN at various applied voltages: (**a**) first cycle and (**b**) 50th cycle.

On the other hand, the methoxy- or *t*-butyl-substituted TPA PAIs **MeO-6d** and ***t*-Bu-6d** exhibited strong absorption at a wavelength around 340 nm in the neutral form, as shown in Figure 7 and Figure S17, respectively. Upon oxidation, two main bands at 338–341 and 779–802 nm appeared, while the main band around 340 nm associated with the neutral state gradually decreased in intensity. The long-wavelength absorption band that appears upon oxidation is characteristic of the TPA cation-radical form. At the same time, the films turned from colorless (**MeO-6d**: L*: 67; a*: 0; b*: 8, ***t*-Bu-6d**: L*: 67; a*: 1; b*: 4) to green (**MeO-6d**: L*: 44; a*: −24; b*: 10, ***t*-Bu-6d**: L*: 56; a*: −8; b*: 1) with the application of increasing voltages. The methoxy- or *t*-butyl-substituted PAIs revealed almost the same absorption profiles and color changes as the initial ones after 50 repeated CV cycles. No orange color was observed. Therefore, substitution at the para position of TPA with methoxy or *t*-butyl can hinder the coupling reaction. All the other **MeO-6** and ***t*-Bu-6** series PAIs showed electrochromic behaviors similar to those of **MeO-6d** and ***t*-Bu-6d**. The enhanced electrochromic performance of these PAIs can be attributed to the substituents, especially the bulky *t*-butyl group, which facilitate the inefficient packing of polymer chains and possibly create micropores in the polymer matrix. In general, for the *t*-butyl-incorporated PAIs, the micropores increased the diffusion rate of the counterions in the polymer matrix, accelerating the response speed, enhancing the coloration efficiency, and showing remarkable switching stability.

### 3.6. Electrochromic Switching Properties

**Figure 8:**
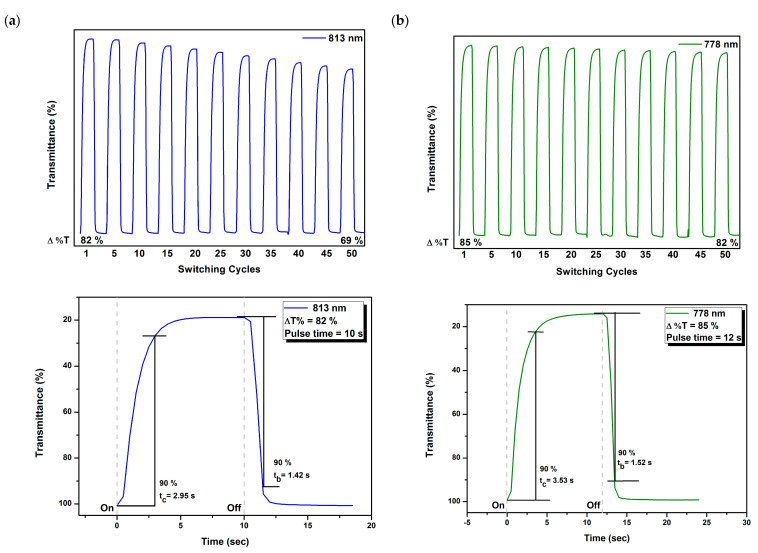
Potential step absorptiometry of the cast film on ITO-glass slide (in MeCN with 0.1 M Bu_4_NClO_4_ as a supporting electrolyte), as measured by application of a potential step: (**a**) optical switching for **6d** at potentials between 0.0 V and 1.05 V and residence time 10 s, monitored at λmax = 813 nm; (**b**) **MeO-6d** at potentials between 0.0 V and 0.85 V and residence time 12 s, monitored at λmax = 778 nm.

Electrochromic switching studies of the polymers were performed by monitoring the % transmittance (%T) as a function of time at their absorption maximum (λ_max_), with response times determined by stepping the potential repeatedly between the neutral and oxidized states. The active area of the polymer film on ITO-glass was approximately 1 cm^2^. As a typical example, Figure 8 depicts the %T changes of PAIs **6d** and **MeO-6d** as a function of time at their respective long-wavelength absorption maxima of 813 nm and 778 nm under square-wave potential steps (between 0 and 1.05 V for **6d** and between 0 and 0.85 V for **MeO-6d**) with residence times of 10 s and 12 s, respectively. The optical contrast, measured as Δ%T, between the neutral and oxidized states was found to be 82% for **6d** and 85% for **MeO-6d** in the first switching cycle. The PAI film of **6d** showed a slight loss of optical contrast after 50 full switches, from 82% to 69%; however, **MeO-6d** showed almost no loss of optical contrast during the first 50 switching cycles.

The response time was calculated at 90% of the full-transmittance change because it is difficult to perceive any further color change with the naked eye beyond this point. As shown in Figure 8a, PAI **6d** attained 90% of a complete color change and bleaching in 2.9 and 1.4 s, respectively. Figure 8b indicates that the PAI **MeO-6d** attained 90% of a complete color change and bleaching in 3.5 and 1.5 s, respectively. The electrochromic coloring efficiency (CE) for the change to blue coloring (ΔOD_813_/Q) of the PAI **6d** was estimated to be 157 cm^2^/C, and that for the change to green coloring (ΔOD_778_/Q) of the PAI **MeO-6d** was estimated to be 195 cm^2^/C. Figure S18 depicts the optical transmittance at 802 nm as a function of time by applying square-wave potential steps between 0 and 0.95 V for a residence time of 15 s for PAI ***t*-Bu-6d.** In general, most of the **MeO-6 and *t*-Bu-6** series PAIs exhibited high electrochromic stability in the first fifty switching cycles. The electrochromic properties of all the polymer films during electro-oxidation processes are summarized in Table 4.

## 4. Conclusions

Three benzamide-containing diamine monomers, 4,4′-bis(*p*-aminobenzamido)triphenylamine (**4**), 4,4′-bis(*p*-aminobenzamido)-4″-methoxytriphenylamine (**MeO-4**), and 4,4′-bis(*p*-aminobenzamido)-4″-*tert*-butyltriphenylamine (***t*-Bu-4**) were synthesized and subsequently used to produce a series of electroactive aromatic PAIs. Insertion of the benzamide spacer between the imide ring and the TPA unit can decrease the oxidation potential and enhance the electrochemical and electrochromic stability of the polymers. For the PAIs derived from diamine monomer **4**, a coupling reaction between TPA units occurred during the oxidative process because there is no protecting substituent at the *p*-position of the TPA pendent phenyl group. In contrast, methoxy or *tert*-butyl-substituted TPA can hinder the coupling reaction in the polymers. The PAIs show high electrochemical and electrochromic stability and strong color changes with high contrast ratios upon electro-oxidation.

## Figures and Tables

**Table 1:** Inherent viscosities and solubility behaviors of PAIs.

Polymer Code	*η*_inh_ (dL/g) ^a^		Solvents ^b,c^					
	PAA	PAI	NMP	DMAc	DMF	DMSO	*m*-Cresol	THF
**6a**	0.69	−	+−	+−	+−	+−	−	−
**6b**	0.91	−	+−	+−	+−	+−	−	−
**6c**	0.67	0.66	+	+	+−	+	+−	−
**6d**	0.50	0.49	+	+	+−	+	+	−
**6e**	0.29	0.35	+	+	+	+	+−	−
**6f**	0.68	0.64	+	+	+	+	+	−
**MeO-6a**	0.74	0.77	+	+	+	+	+-	−
**MeO-6b**	0.87	0.88	+	+	+-	+	+-	−
**MeO-6c**	0.58	0.58	+	+	+	+	+	−
**MeO-6d**	0.70	0.70	+	+	+-	+	+	−
**MeO-6e**	0.59	0.57	++	++	++	++	+	−
**MeO-6f**	0.92	0.56	++	++	++	++	+	+−
***t*** **-Bu-6a**	0.40	0.80	+	+	+	+	+	−
***t*** **-Bu-6b**	0.45	0.65	+	+	+	+	+	−
***t*** **-Bu-6c**	0.65	0.64	+	+	+	+	+	−
***t*** **-Bu-6d**	0.39	0.58	+	+	+	+	+	−
***t*** **-Bu-6e**	0.46	0.43	++	++	++	++	+	−
***t*** **-Bu-6f**	0.48	0.50	++	++	++	++	+	+−

^a^ Inherent viscosity measured at a concentration of 0.5 dL/g in DMAc at 30 °C. ^b^ The qualitative solubility was tested with 10 mg of a sample in 1 mL of stirred solvent. ++: soluble at room temperature; +: soluble on heating; +−: partially soluble; −: insoluble even on heating. ^c^ Solvent: NMP: N-methyl-2-pyrrolidone; DMAc: N,N-dimethylacetamide; DMF: N,N-dimethylformamide; DMSO: dimethyl sulfoxide; THF: tetrahydrofuran.

**Table 2:** Thermal properties of PAIs.

Polymer Code	*T*_g_ (°C) ^a^	*T*_d_ at 5% Weight Loss (°C) ^b^		*T*_d_ at 10% Weight Loss (°C) ^b^		Char Yield (wt %) ^c^
		N_2_	Air	N_2_	Air	
**6a**	355	525	537	553	575	58
**6b**	326	545	550	578	600	60
**6c**	304	532	539	576	589	55
**6d**	296	504	488	541	549	58
**6e**	331	496	514	523	559	58
**6f**	326	549	535	585	576	62
**MeO-6a**	325	464	438	495	469	68
**MeO-6b**	323	483	482	519	520	66
**MeO-6c**	299	500	476	532	522	50
**MeO-6d**	286	501	473	539	509	60
**MeO-6e**	300	444	447	562	478	52
**MeO-6f**	326	480	473	522	511	55
***t*** **-Bu-6a**	350	527	515	560	554	58
***t*** **-Bu-6b**	304	513	522	549	556	58
***t*** **-Bu-6c**	309	479	473	512	522	55
***t*** **-Bu-6d**	289	501	509	532	554	56
***t*** **-Bu-6e**	313	423	437	448	471	50
***t*** **-Bu-6f**	322	511	486	552	525	58

^a^ Midpoint temperature of the baseline shift on the second DSC heating trace (rate = 20 °C/min) after quenching from 400 to 50 °C in nitrogen. ^b^ Decomposition temperature at which a 5% or 10% weight loss was recorded by TGA at a heating rate of 20 °C/min and a gas flow rate of 20 cm^3^/min. ^c^ Residual weight % at 800 °C at a scan rate 20 °C/min in nitrogen.

**Table 3:** Optical and electrochemical properties of the polymers.

Polymer Code	Thin Film (nm)		Oxidation Potential (V) ^a^		*E*_g_^opt^(eV) ^b^	HOMO (eV) ^c^	LUMO (eV) ^d^
	λ_max_	λ_onset_	*E*_onset_	*E*_1/2_^Ox^			
**6a**	330	420	0.70	0.84	2.95	5.20	2.25
**6b**	338	407	0.71	0.83	3.05	5.19	2.14
**6c**	346	418	0.70	0.83	2.97	5.19	2.22
**6d**	341	411	0.70	0.82	3.02	5.18	2.16
**6e**	344	409	0.70	0.82	3.03	5.18	2.15
**6f**	342	406	0.72	0.83	3.05	5.19	2.14
**MeO-6a**	343	425	0.56	0.71	2.92	5.07	2.15
**MeO-6b**	344	413	0.59	0.71	3.00	5.07	2.07
**MeO-6c**	347	422	0.59	0.72	2.94	5.08	2.14
**MeO-6d**	341	425	0.59	0.72	2.92	5.08	2.16
**MeO-6e**	348	425	0.59	0.71	2.92	5.07	2.15
**MeO-6f**	346	419	0.59	0.72	2.96	5.08	2.12
***t*** **-Bu-6a**	333	421	0.67	0.82	2.94	5.18	2.24
***t*** **-Bu-6b**	336	406	0.64	0.78	3.05	5.14	2.09
***t*** **-Bu-6c**	332	421	0.63	0.77	2.95	5.13	2.18
***t*** **-Bu-6d**	339	414	0.68	0.80	3.00	5.16	2.16
***t*** **-Bu-6e**	341	421	0.65	0.78	2.95	5.14	2.19
***t*** **-Bu-6f**	345	413	0.66	0.79	3.00	5.15	2.15

^a^ vs. Ag/AgCl in CH_3_CN. *E*_1/2_ = average potential of redox couple peaks. ^b^ Bandgap calculated from absorption edge of the polymer film. Energy gap = 1240/λ_onset._^c^ The HOMO energy levels were calculated from *E*_1/2_^Ox^ values of CV curves and were referenced to ferrocene (4.8 eV relative to the vacuum energy level). ^d^ LUMO = HOMO − *E*_g_^opt^.

**Table 4:** Electrochromic properties of PAIs.

Polymer Code	λ_max_ ^a^ (nm)	Δ%T	Response Time ^b^		ΔOD ^c^	Q_d_ ^d^ (mC/cm^2^)	CE ^e^ (cm^2^/C)
			t_c_ (s)	t_b_ (s)			
**6a**	804	77	3.7	2.5	1.35	16.42	82
**6b**	818	73	4.8	2.3	0.85	9.29	91
**6c**	818	52	2.9	1.1	0.33	1.82	182
**6d**	813	82	3.0	1.4	0.73	4.63	157
**6e**	810	56	3.2	1.0	0.38	2.85	131
**6f**	809	89	6.3	3.2	1.36	9.36	145
**MeO-6a**	779	74	4.4	2.0	0.88	6.71	131
**MeO-6b**	779	69	2.6	1.4	0.77	5.07	153
**MeO-6c**	780	80	5.4	3.1	0.63	4.55	140
**MeO-6d**	779	85	3.5	1.5	0.74	4.34	195
**MeO-6e**	780	86	3.2	1.7	1.06	7.17	148
**MeO-6f**	775	79	2.2	3.0	1.31	9.65	136
**t-Bu-6a**	791	97	2.7	2.0	1.63	10.39	157
**t-Bu-6b**	779	88	3.2	1.6	0.92	5.23	176
**t-Bu-6c**	799	77	3.1	1.4	0.63	4.63	136
**t-Bu-6d**	802	88	3.1	1.4	0.96	6.62	145
**t-Bu-6e**	795	72	2.9	1.4	0.57	4.42	128
**t-Bu-6f**	796	62	3.1	1.2	1.38	10.86	150

^a^ Wavelength of absorption maximum. ^b^ Time to 90% of the full-transmittance change. ^c^ Optical density (ΔOD) = log[T_bleached_/T_colored_], where T_colored_ and T_bleached_ are the maximum transmittances in the oxidized and neutral states, respectively. ^d^ Q_d_ is ejected charge, asdetermined from the in situ experiments. ^e^ Coloration efficiency (CE) = ΔOD/Q_d_.

## Data Availability

The original contributions presented in this study are included in the article. Further inquiries can be directed to the corresponding author.

## Supplementary Materials

The following supplemental materials are available online at https://www.mdpi.com/article/10.3390/polym17091152/s1: Materials and instrumentation, FTIR, ^1^H NMR, and ^13^C NMR spectra of the prepared monomers and polymers, thermal properties, CV and spectroelectrochemical diagrams of the prepared polymer films. Figure S1: IR spectra of diamine monomer 4 and its precursor compounds. Figure S2: IR spectra of diamine monomers MeO-4 and t-Bu-4 and their precursor compounds. Figure S3: 1H NMR, 13C NMR, H-H COSY, and C-H HMQC NM spectra of diamide-dinitro compound 3 in DMSO-d6. Figure S4: 1H NMR, 13C NMR, H-H COSY, and C-H HMQC NMR spectra of diamide-diamine 4 in DMSO-d6. Figure S5: 1H NMR, 13C NMR, H-H COSY, and C-H HMQC NMR spectra of diamide-dinitro compound MeO-3 in DMSO-d6. Figure S6: 1H NMR, 13C NMR, H-H COSY, and C-H HMQC NMR spectra of diamide-diamine MeO-4 in DMSO-d6. Figure S7: 1H NMR spectra of diamide-dinitro compound t-Bu-3 and diamide-diamine t-Bu-4 in DMSO-d6. Figure S8: IR spectra of PAI 6f and its poly(amide-amic acid) precursor. Figure S9: Proton NMR spectra of (a) PAI 6f, (b) PAI MeO-6f, and (c) PAI t-Bu-6f in DMSO-d6. Figure S10: DSC curves of the 6 series PAIs and typical TGA curves of PAI 6f. Figure S11: CV scans of the cast films of PAIs (a) 6a, (b) 6b, (c) 6c, (d) 6d, (e) 6e, and (f) 6f on an ITO-coated glass substrate in 0.1 M Bu4NClO4/MeCN solutions at a scan rate of 50 mV/s. Figure S12: Repetitive CV scans of the cast films of PAIs (a) 6a, (b) 6b, (c) 6c, (d) 6d, (e) 6e, and (f) 6f on an ITO-coated glass substrate in 0.1 M Bu4NClO4/MeCN solutions at a scan rate of 50 mV/s. Figure S13: CV scans of the cast films of PAIs (a) MeO-6a, (b) MeO-6b, (c) MeO-6c, (d) MeO-6d, (e) MeO-6e, and (f) MeO-6f on an ITO-coated glass substrate in 0.1 M Bu4NClO4/MeCN solutions at a scan rate of 50 mV/s. Figure S14: Repetitive CV scans of the cast films of PAIs (a) MeO-6a, (b) MeO-6b, (c) MeO-6c, (d) MeO-6d, (e) MeO-6e, and (f) MeO-6f on an ITO-coated glass substrate in 0.1 M Bu4NClO4/MeCN solutions at a scan rate of 50 mV/s. Figure S15: CV scans of the cast films of PAIs (a) t-Bu-6a, (b) t-Bu-6b, (c) t-Bu -6c, (d) t-Bu-6d, (e) t-Bu-6e, and (f) t-Bu-6f on an ITO-coated glass substrate in 0.1 M Bu4NClO4/MeCN solutions at a scan rate of 50 mV/s. Figure S16: Repetitive CV scans of the cast films of PAIs (a) t-Bu-6a, (b) t-Bu-6b, (c) t-Bu-6c, (d) t-Bu-6d, (e) t-Bu-6e, and (f) t-Bu-6f on an ITO-coated glass substrate in 0.1 M Bu4NClO4/MeCN solutions at a scan rate of 50 mV/s. Figure S17: Spectroelectrograms and color changes of PAI t-Bu-6d on an ITO-glass slide in 0.1 M Bu4NClO4/MeCN at various applied voltages (a) first cycle and (b) 50th cycle. Figure S18: Potential step absorptiometry of the cast film PAI t-Bu-6d of on ITO-glass slide (in MeCN with 0.1 M Bu4NClO4 as a supporting electrolyte) by applying a potential step between 0 and 0.95 V for a resident time of 15 s at 802 nm.
