# Supplementary material for: Synthesis and Electrochromic Properties of Triphenylamine-Based Aromatic Poly(amide-imide)s
Source: Polymers (Basel). 2025 Apr 23;17(9):1152. doi: 10.3390/polym17091152 (PMC12073670; doi:10.3390/polym17091152)
Supplement: Supplementary file 1 [file polymers-17-01152-s001.zip › polymers-3575205-supplementary.pdf]

<u>**Supplementary Materials**</u>

Synthesis and Electrochromic Properties of Triphenylamine-Based Aromatic Poly(amide-imide)s

Sheng-Huei Hsiao* and Zong-De Ni
Department of Chemical Engineering, National Taipei University of Technology, Taipei, Taiwan *Correspondence: shhsiao@ntut.edu.tw (S.H. Hsiao)

Materials and Measurements

Aniline (Acros), *p*-anisidine (Acros), *p*-fluoronitrobenzene (Acros), *p-tert*-butylaniline (AKSci), *p*-nitrobenzoyl chloride (Acros), cesium fluoride (CsF, Acros), hydrazine monohydrate (Alpha), 10% palladium on activated carbon (Pd/C, Fluka), dimethyl sulfoxide (DMSO, Tedia), and acetic anhydride (Acros) were used as received from commercial sources. *N*-Methyl-2-pyrrolidone (NMP, Tedia) was dried over calcium hydride for 24 h, distilled under reduced pressure, and stored over 4 Å molecular sieves in sealed bottles. The aromatic tetracarboxylic dianhydrides including pyromellitic dianhydride (PMDA; **5a**, TCI), 3,3',4,4'-biphenyltetracarboxylic dianhydride (BPDA; **5b** Oxychem), 3,3',4,4'-benzophenonetetracarboxylic dianhydride (BTDA; **5c** Oxychem), 4,4'-oxydiphthalic anhydride (ODPA; **5d**, Oxychem), 3,3',4,4'-diphenylsulfonetetracarboxylic dianhydride (DSDA; **5e**, Oxychem), 2,2-bis(3,4-dicarboxyphenyl)hexafluoropropane dianhydride (6FDA; **5f**, Hoechst Celanese) were purified by dehydration at 250 ^{o}C in vacuum for 3 h. Other reagents and solvents were used as received from commercial sources. According to a reported synthetic procedure [14], 4,4'-diaminotriphenylamine (**2**), 4,4'-diamino-4"-methoxytriphenylamine (**MeO-2**), and 4,4'-diamino-4"-*tert*-butyltriphenylamine (***t*-Bu-2**) were synthesized via the fluoro-displacement of *p*-fluoronitrobenzene with aniline, *p*-anisidine and *p-tert*-butylaniline in the presence of CsF in DMSO, followed by Pd/C-catalyzed hydrazine reduction of the intermediate dinitro compounds 4,4'-dinitrotriphenylamine (**1**), 4-methoxy-4',4"-dinitrotriphenylamine (**MeO-1**), and 4-*tert*-butyl-4',4"-dinitrotriphenylamine (***t*-Bu-1**) in ethanol, respectively.

Infrared (IR) spectra were recorded on a Horiba FT-720 FT-IR spectrometer. ^{1}H spectra were measured on a Bruker Avance III HD-600 MHz NMR spectrometer. Elemental analyses were run in a Heraeus VarioEL III CHNS elemental analyzer. The inherent viscosities were determined with a Cannon-Fenske viscometer at 30 ^{o}C. Gel permeation chromatography (GPC) analysis was carried out on a Waters chromatography unit interfaced with a Waters 2410 refractive index detector at 3 mg/mL concentration. Two Waters 5 μm Styragel HR-2 and HR-4 columns (7.8 mm I. D. x 300 mm) were connected in series with NMP as the eluent at a flow rate of 0.6

mL/min at 50 ^{o}C and were calibrated with polystyrene standards. Thermogravimetric analysis (TGA) was performed with a Perkin-Elmer Pyris 1 TGA. Experiments were carried out on approximately 3−5 mg of polymer film samples heated in flowing nitrogen or air (flow rate = 40 cm^3/min) at a heating rate of 20 ^{o}C/min. DSC analyses were performed on a Perkin-Elmer DSC 4000 at a scan rate of 20 ^{o}C/min in flowing nitrogen. Electrochemistry was performed with a CHI 750A electrochemical analyzer. Voltammograms are presented with the positive potential pointing to the left and with increasing anodic currents pointing downwards. Cyclic voltammetry was conducted with the use of a three-electrode cell in which ITO (polymer films area about 0.5 cm × 2.0 cm) was used as a working electrode. All cell potentials were taken with the use of a home-made Ag/AgCl, KCl (sat.) reference electrode. Ferrocene was used as an external reference for calibration (+0.44 V vs. Ag/AgCl). Spectroelectrochemistry analyses were carried out with an electrolytic cell, which was composed of a 1 cm cuvette, ITO as a working electrode, a platinum wire as an auxiliary electrode, and a home-made Ag/AgCl, KCl (sat.) reference electrode. Absorption spectra in the spectroelectrochemical experiments were also measured with an Agilent 8453 UV-visible photodiode array spectrophotometer.

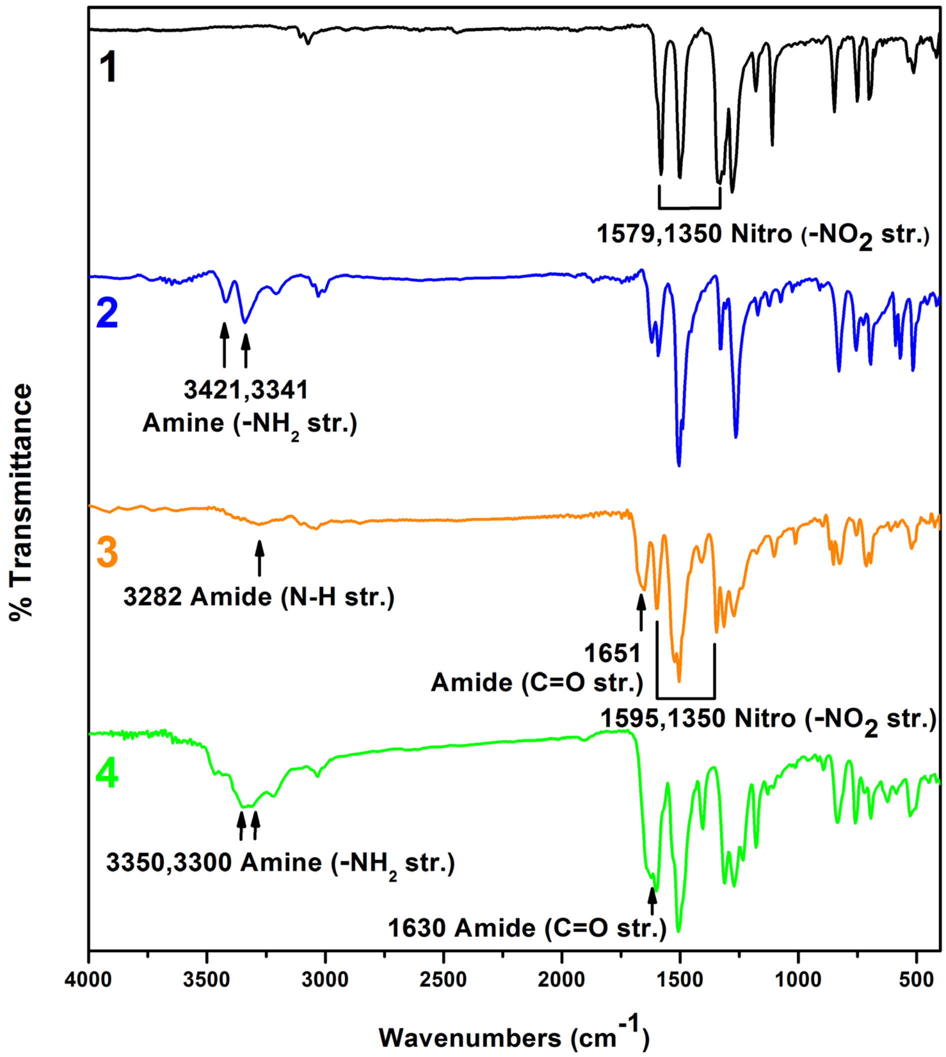

Figure S1. IR spectra of diamine monomer **4** and its precursor compounds.

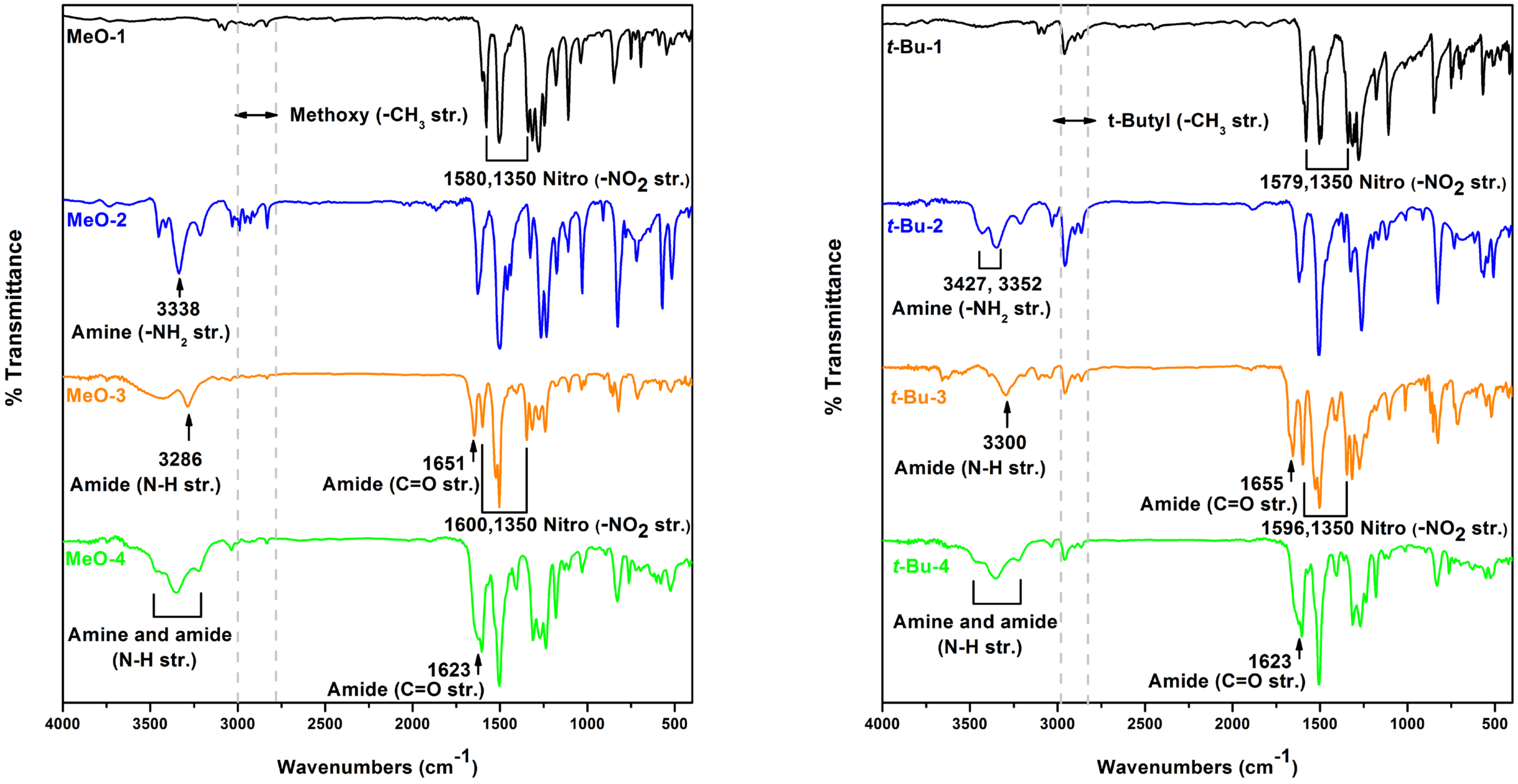

Figure S2. IR spectra of diamine monomers **MeO-4** and ***t*-Bu-4** and their precursor compounds.

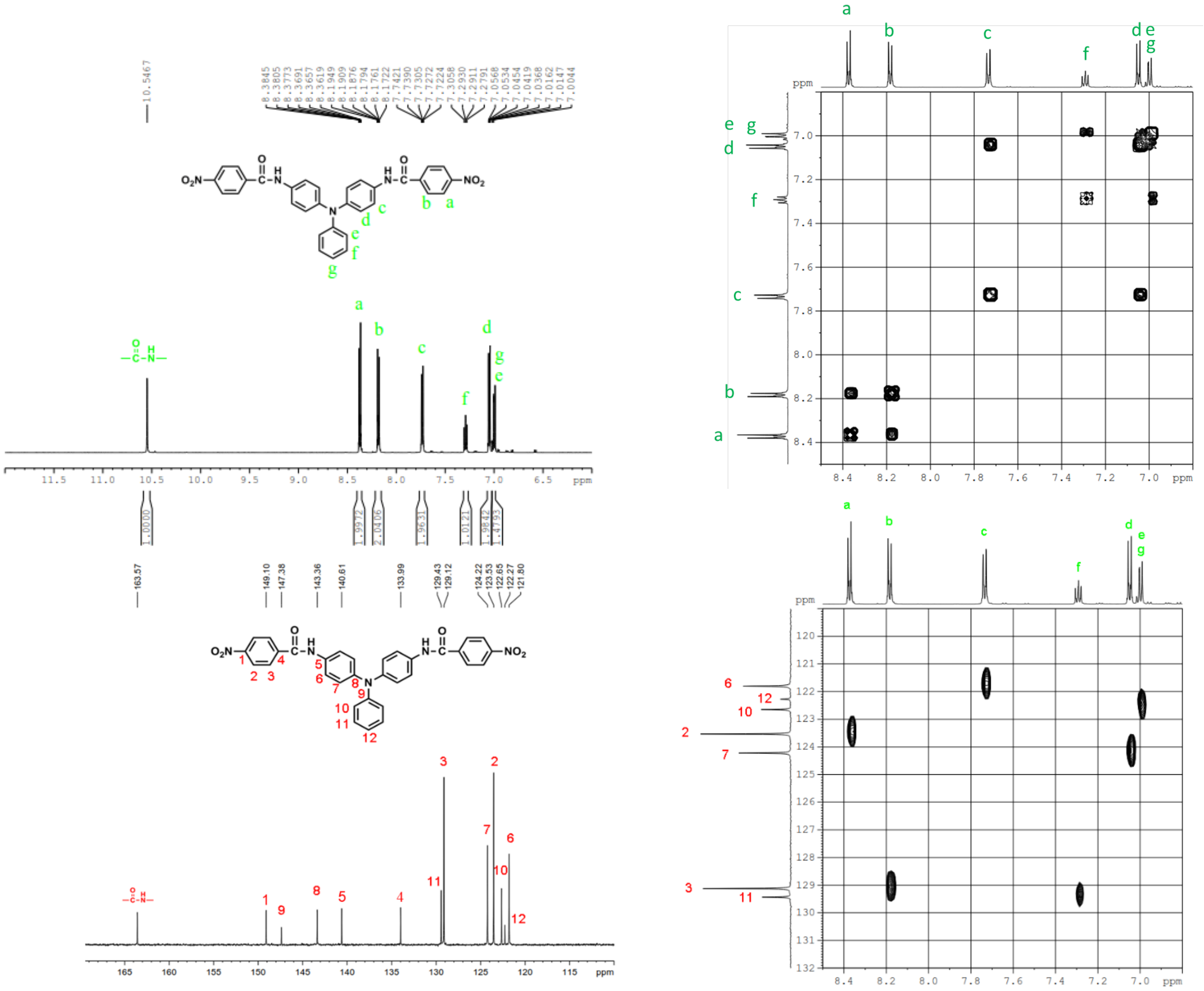

Figure S1. ^{1}H NMR, ^{13}C NMR, H-H COSY, and C-H HMQC NMR spectra of diamide-dinitro compound **3** in DMSO-d_6.

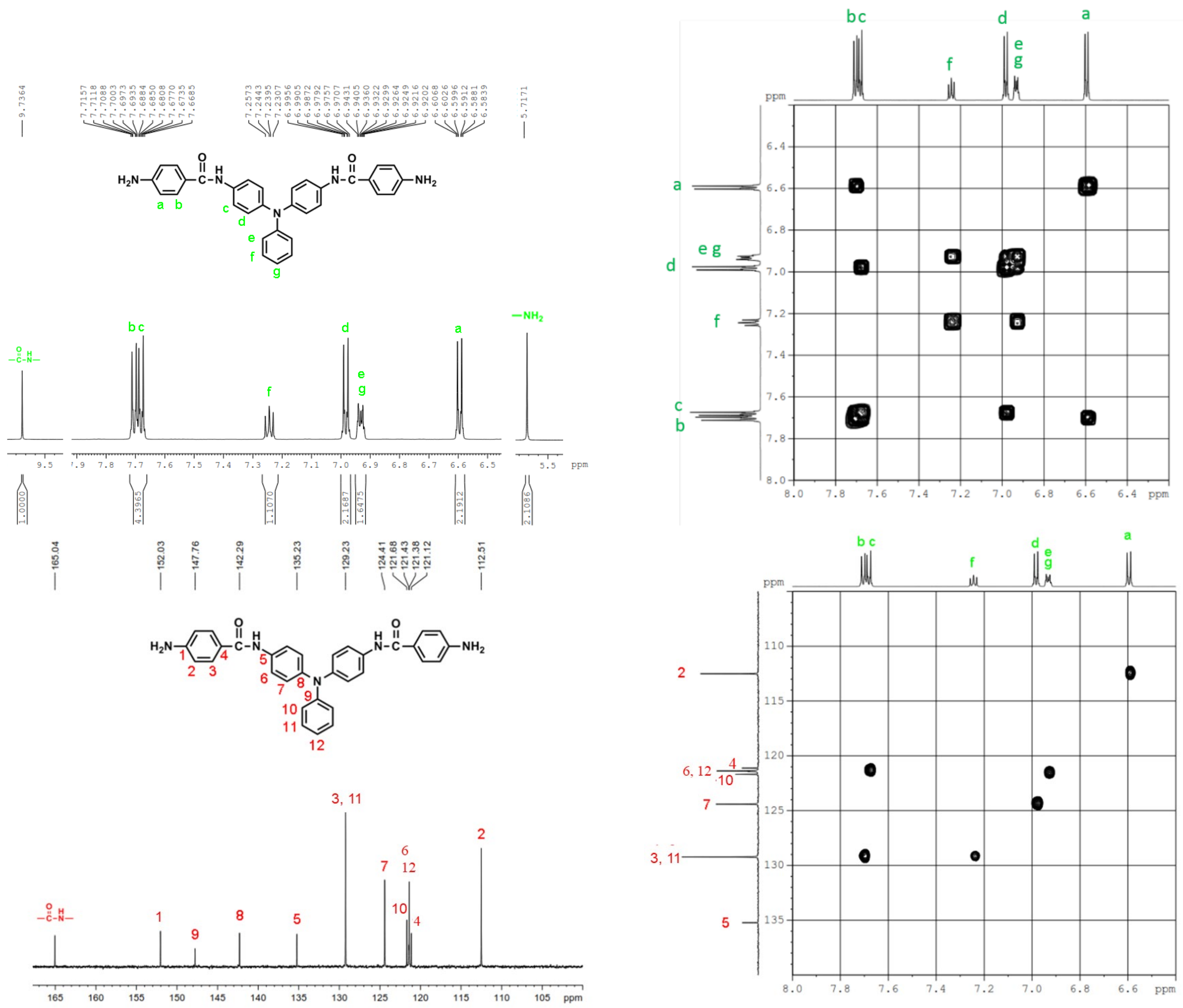

Figure S4. ^{1}H NMR, ^{13}C NMR, H-H COSY, and C-H HMQC NMR spectra of diamide-diamine **4** in DMSO-d_6.

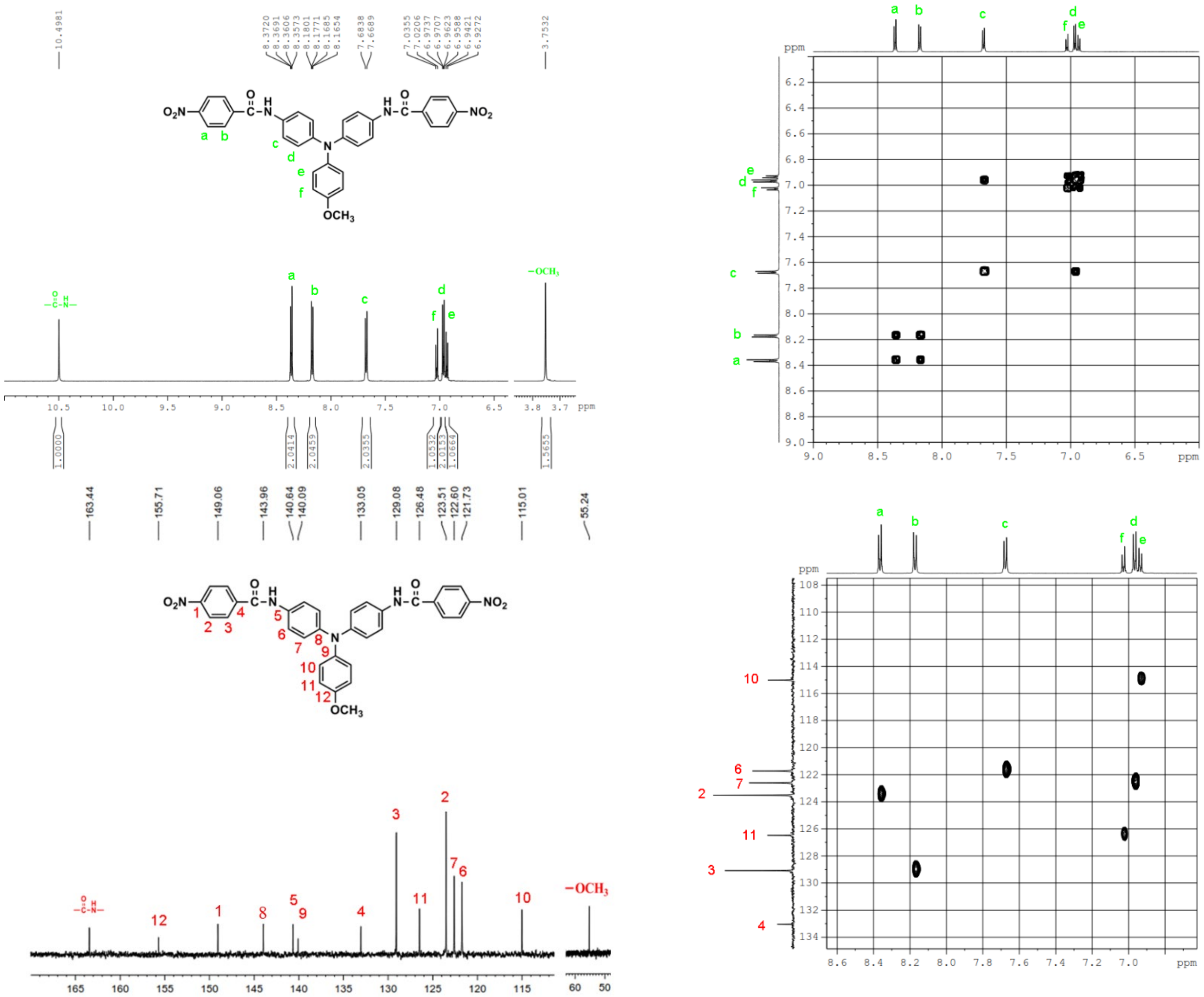

Figure S5. ^{1}H NMR, ^{13}C NMR, H-H COSY, and C-H HMQC NMR spectra of diamide-dinitro compound **MeO-3** in DMSO-d_6.

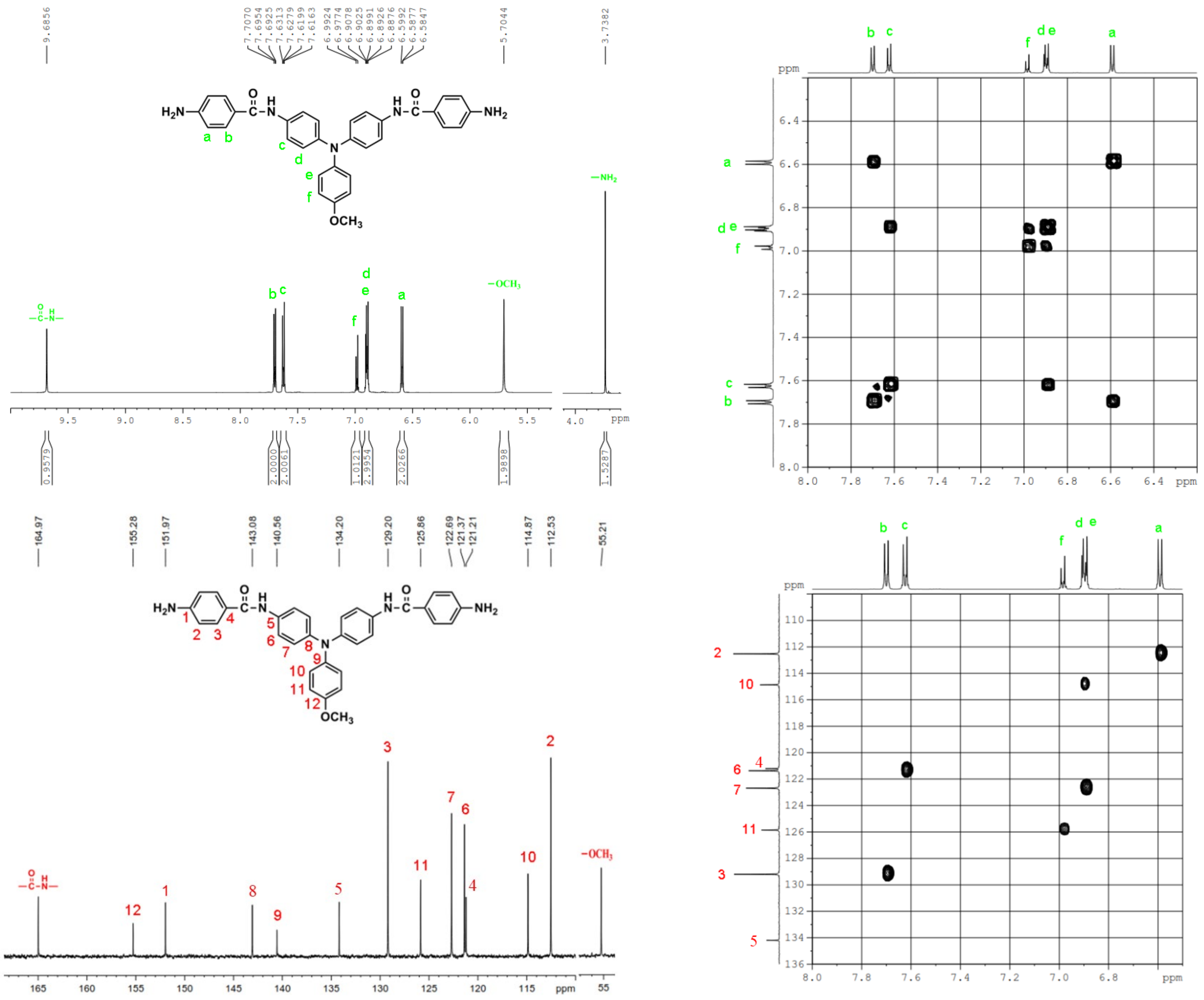

Figure S6. ^{1}H NMR, ^{13}C NMR, H-H COSY, and C-H HMQC NMR spectra of diamide-diamine **MeO-4** in DMSO-d_6.

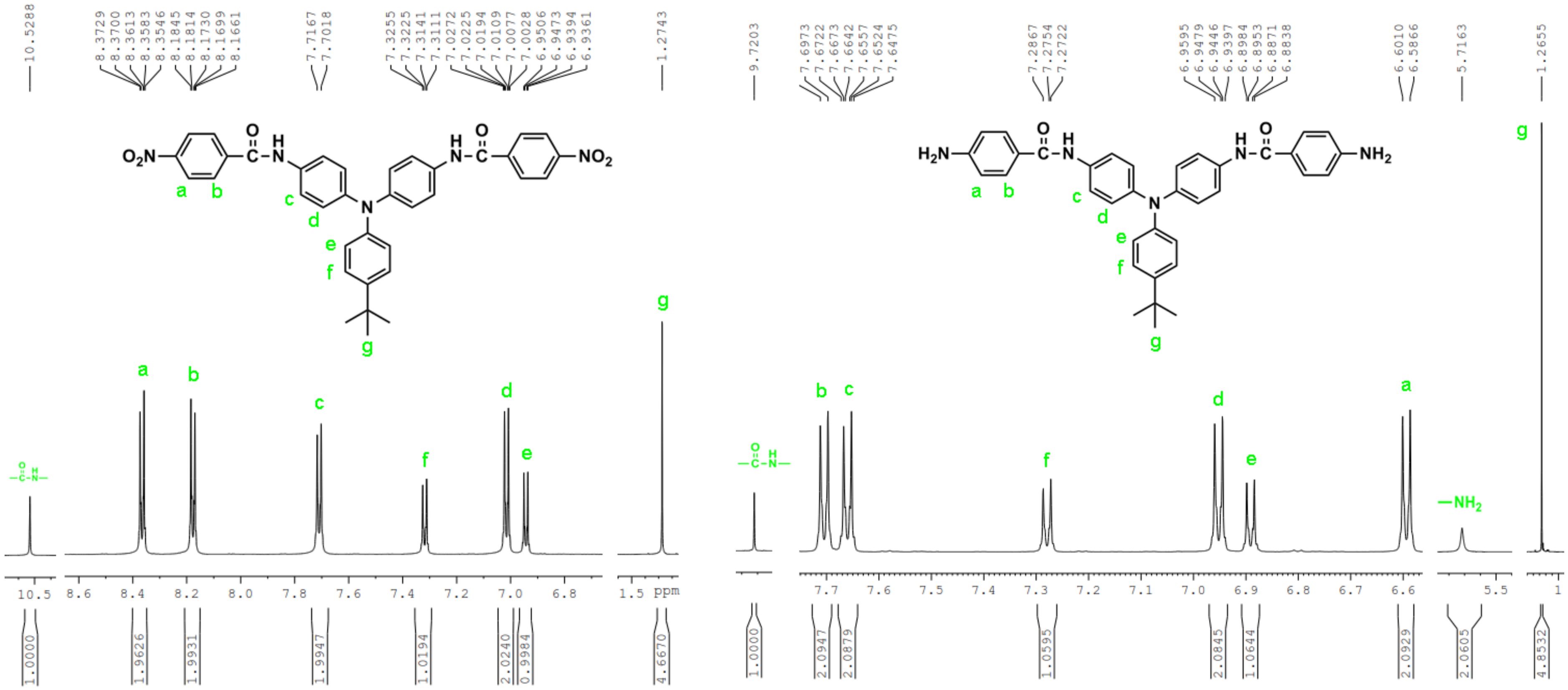

Figure S7. ^{1}H NMR spectra of diamide-dinitro compound ***t*-Bu-3** and diamide-diamine ***t*-Bu-4** in DMSO-d_6.

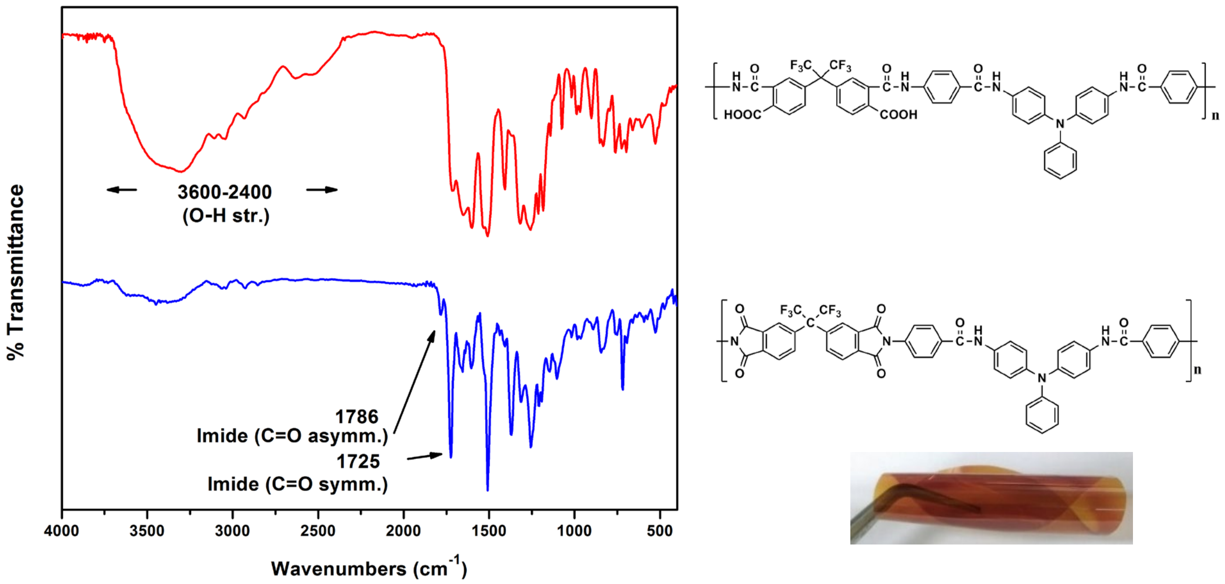

Figure S8. IR spectra of PAI **6f** and its poly(amide-amic acid) precursor.

(a)

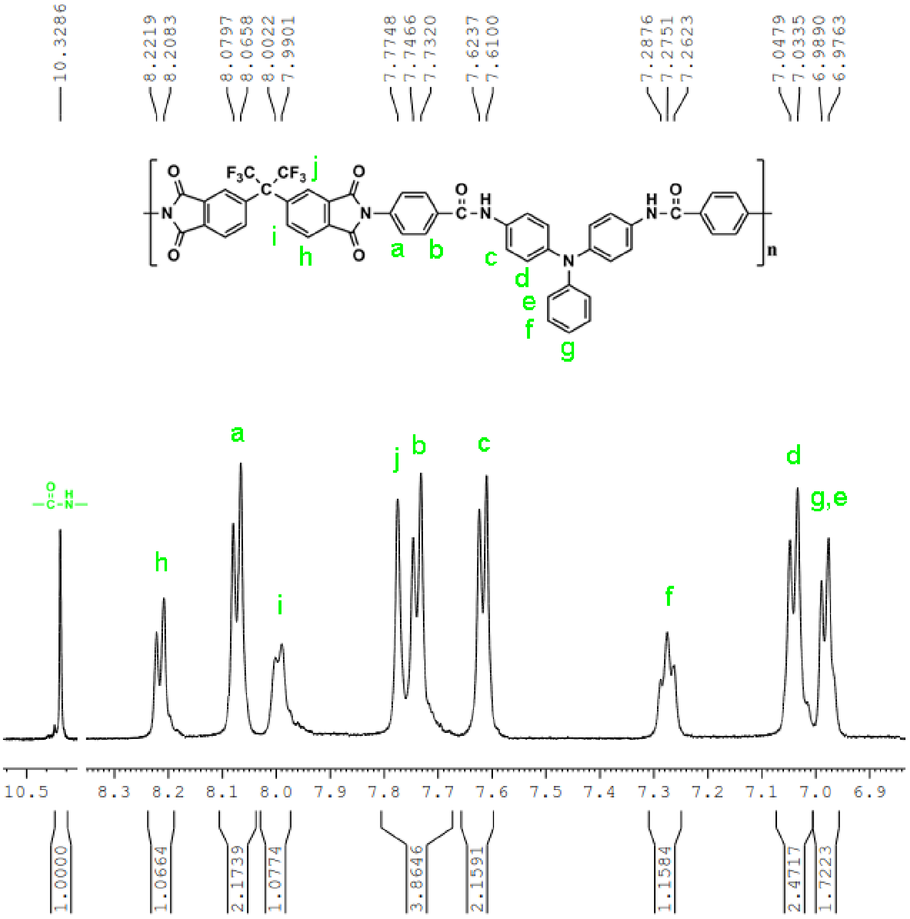

Figure S9. (a) Proton NMR spectrum of PAI **6f** in DMSO-d_6.

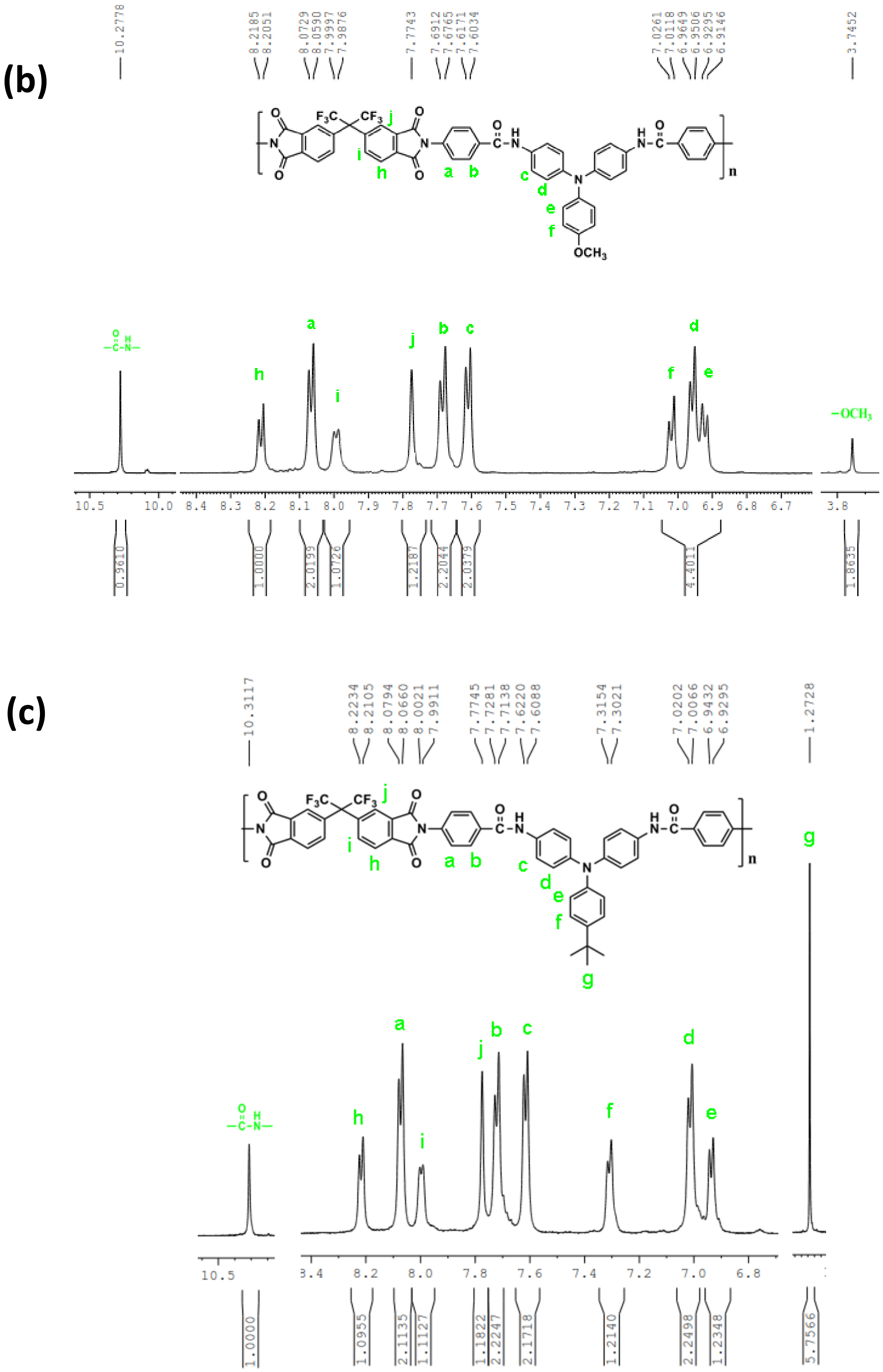

Figure S9. Proton NMR spectra of (b) PAI **MeO-6f** and (c) PAI ***t*-Bu-6f** in DMSO-d_6.

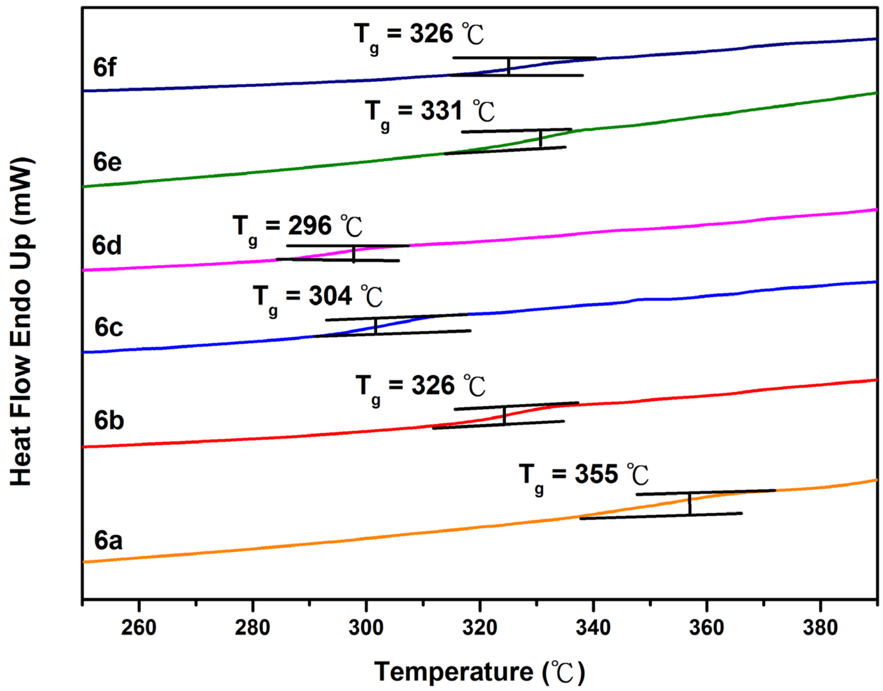

Figure S10. DSC curves of the **6** series PAIs.

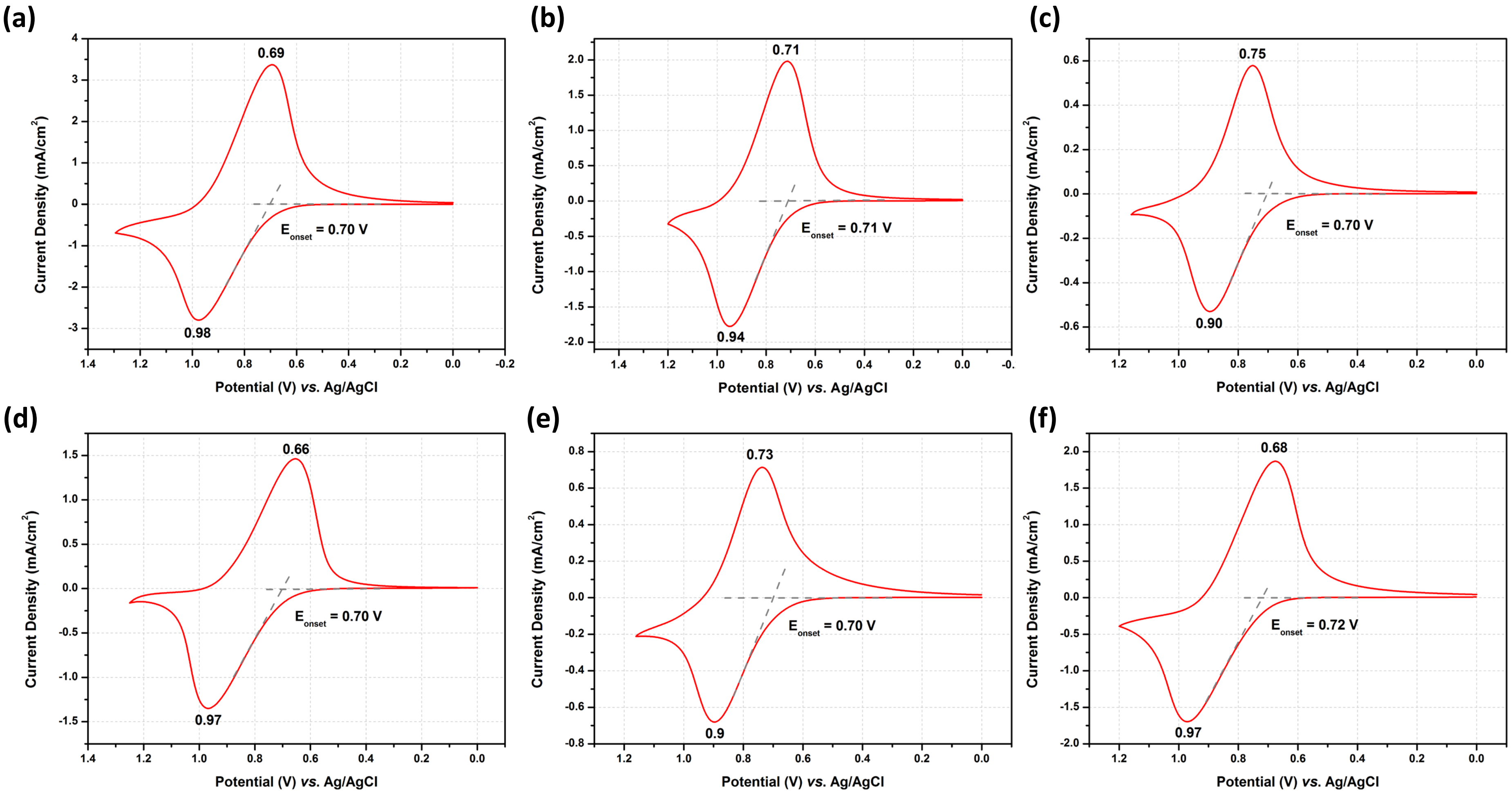

Figure S11. CV scans of the cast films of PAIs (a) **6a**, (b) **6b**, (c) **6c,** (d) **6d**, (e) **6e**, and (f) **6f** on an ITO-coated glass substrate in 0.1 M Bu_4NClO_4/MeCN solutions at a scan rate of 50 mV/s.

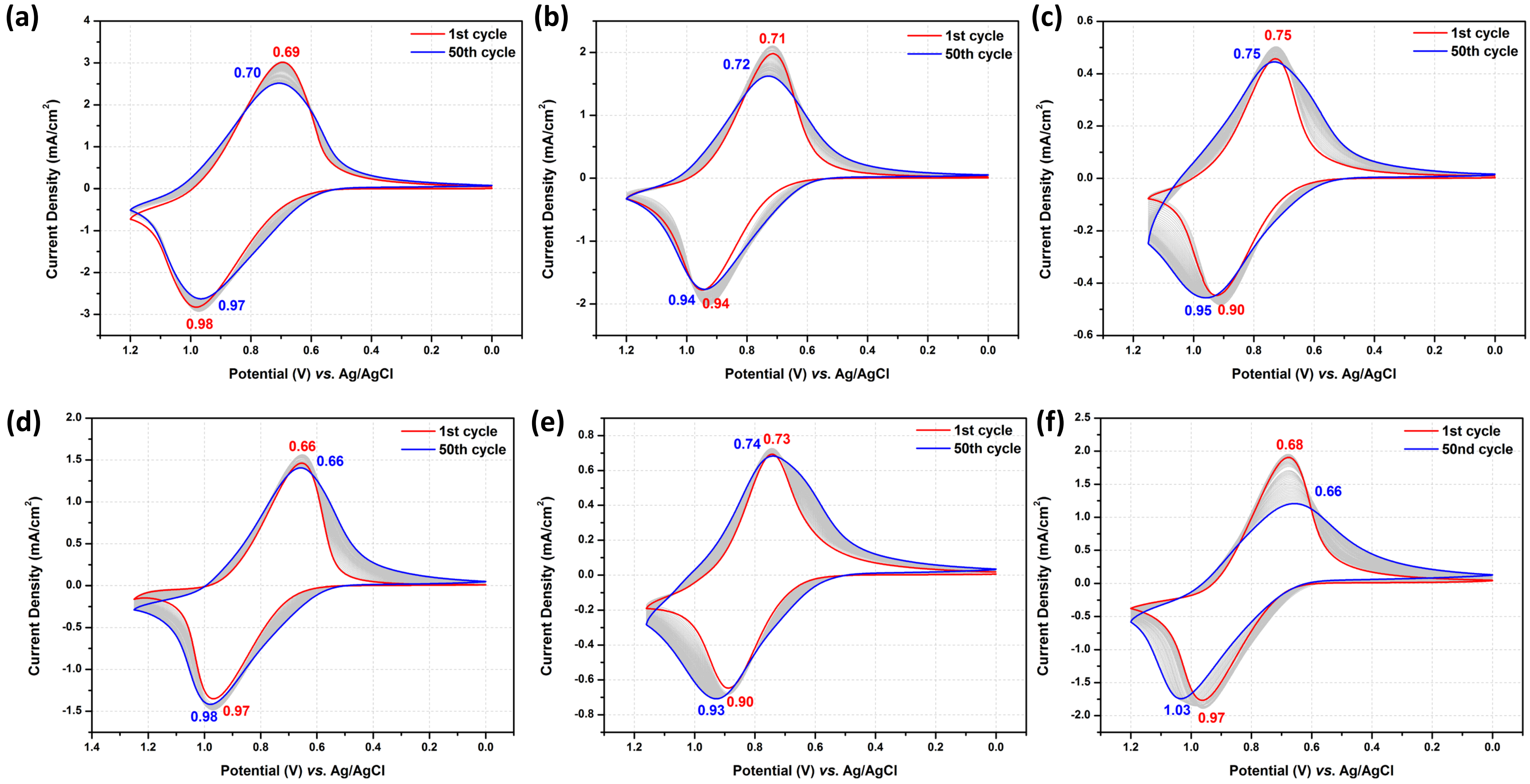

Figure S12. Repetitive CV scans of the cast films of PAIs (a) **6a**, (b) **6b**, (c) **6c,** (d) **6d**, (e) **6e**, and (f) **6f** on an ITO-coated glass substrate in 0.1 M Bu_4NClO_4/MeCN solutions at a scan rate of 50 mV/s.

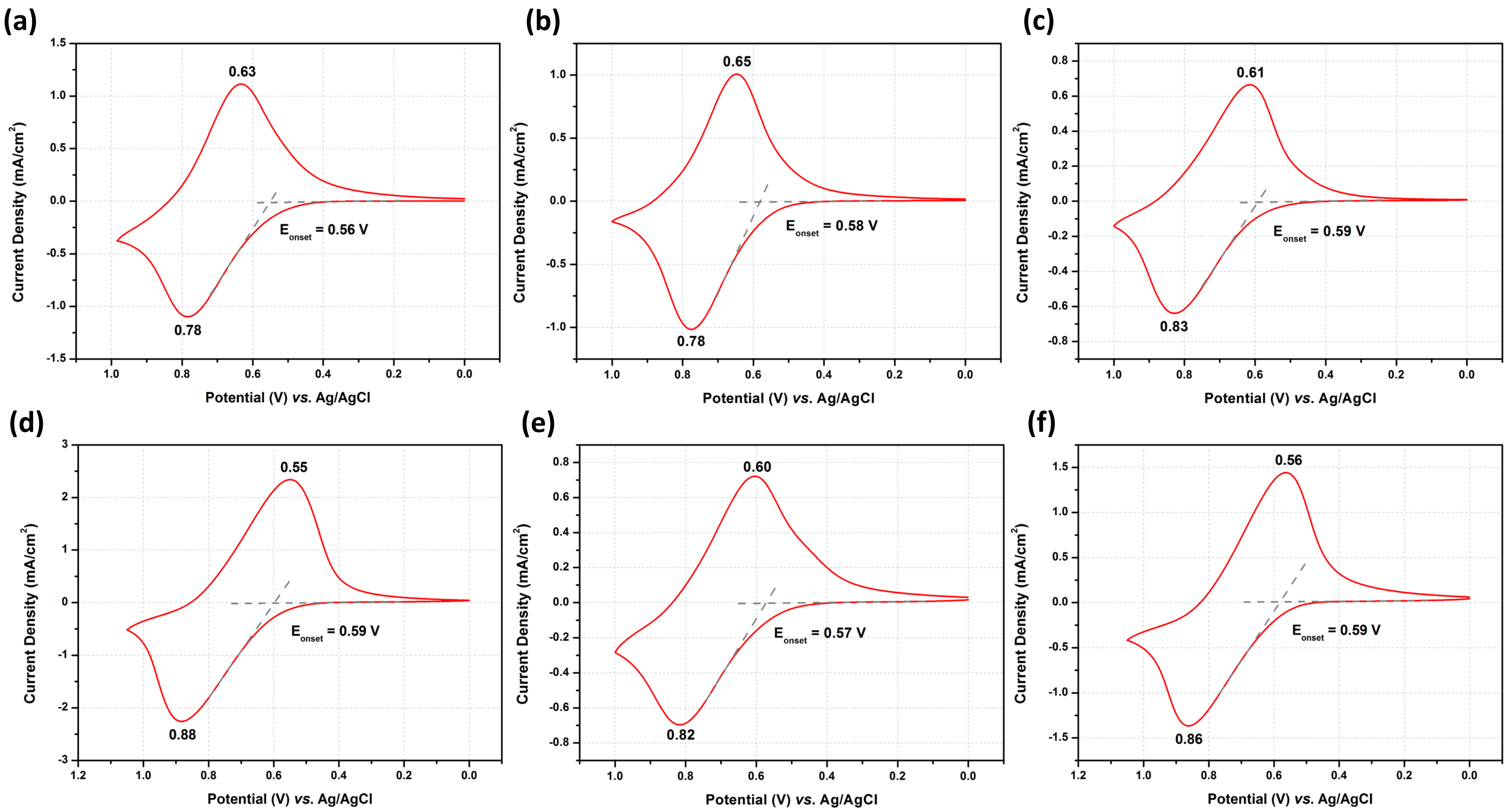

Figure S13. CV scans of the cast films of PAIs (a) **MeO-6a**, (b) **MeO-6b**, (c) **MeO-6c,** (d) **MeO-6d**, (e) **MeO-6e**, and (f) **MeO-6f** on an ITO-coated glass substrate in 0.1 M Bu_4NClO_4/MeCN solutions at a scan rate of 50 mV/s.

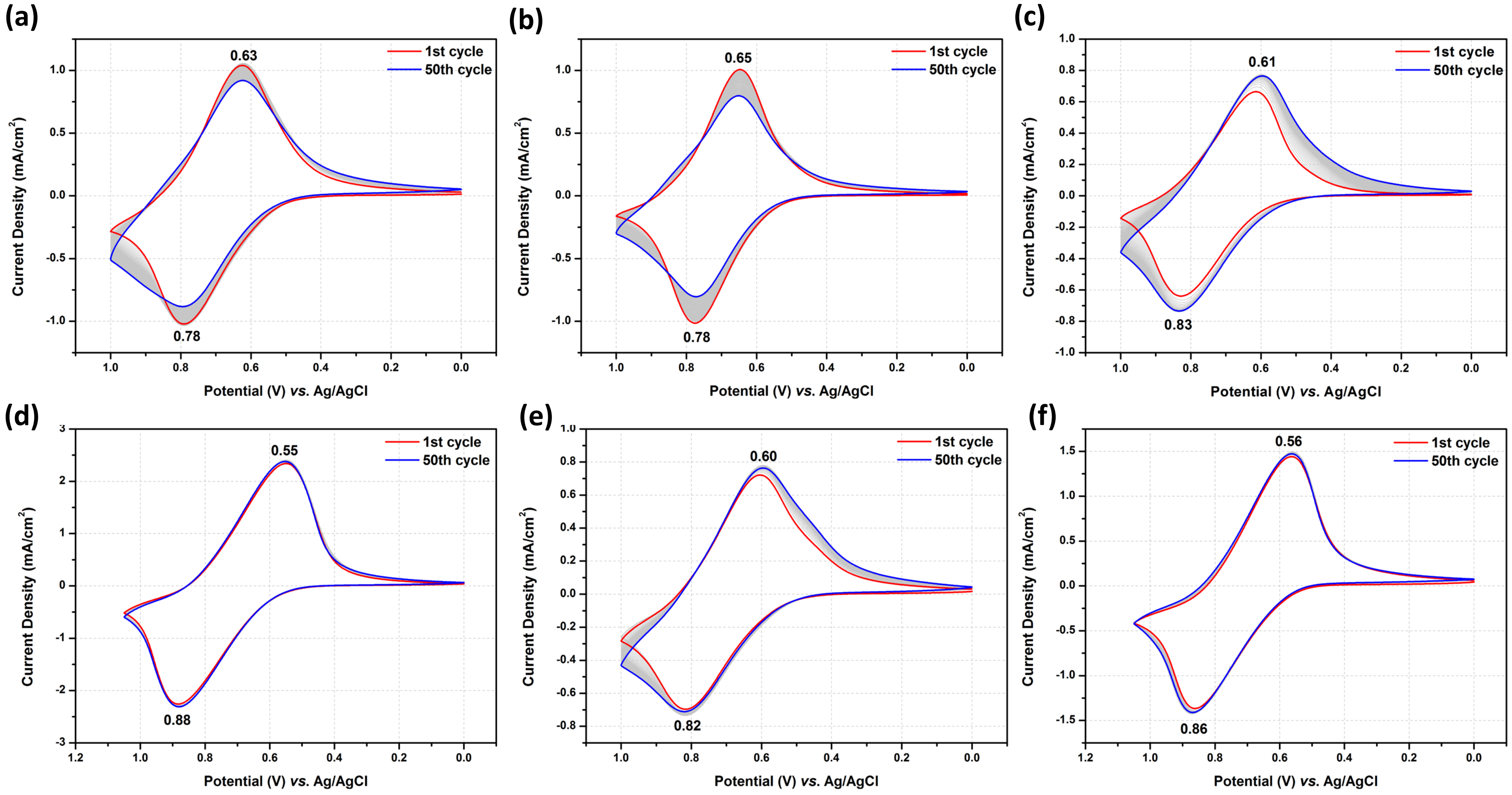

Figure S14. Repetitive CV scans of the cast films of PAIs (a) **MeO-6a**, (b) **MeO-6b**, (c) **MeO-6c,** (d) **MeO-6d**, (e) **MeO-6e**, and (f) **MeO-6f** on an ITO-coated glass substrate in 0.1 M Bu_4NClO_4/MeCN solutions at a scan rate of 50 mV/s.

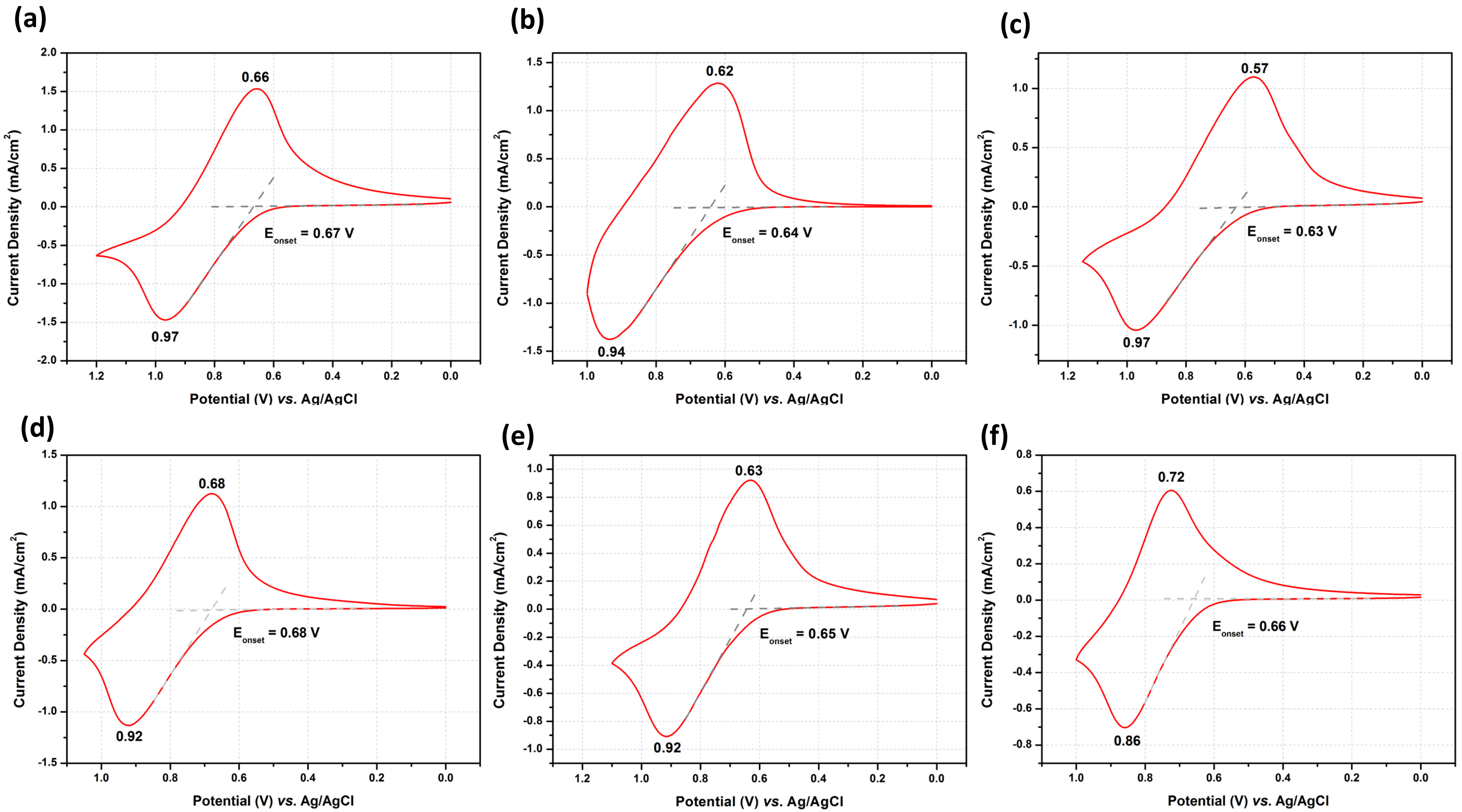

Figure S15. CV scans of the cast films of PAIs (a) ***t*-Bu-6a**, (b) ***t*-Bu-6b**, (c) ***t*-Bu -6c,** (d) ***t*-Bu-6d**, (e) ***t*-Bu-6e**, and (f) ***t*-Bu-6f** on an ITO-coated glass substrate in 0.1 M Bu_4NClO_4/MeCN solutions at a scan rate of 50 mV/s.

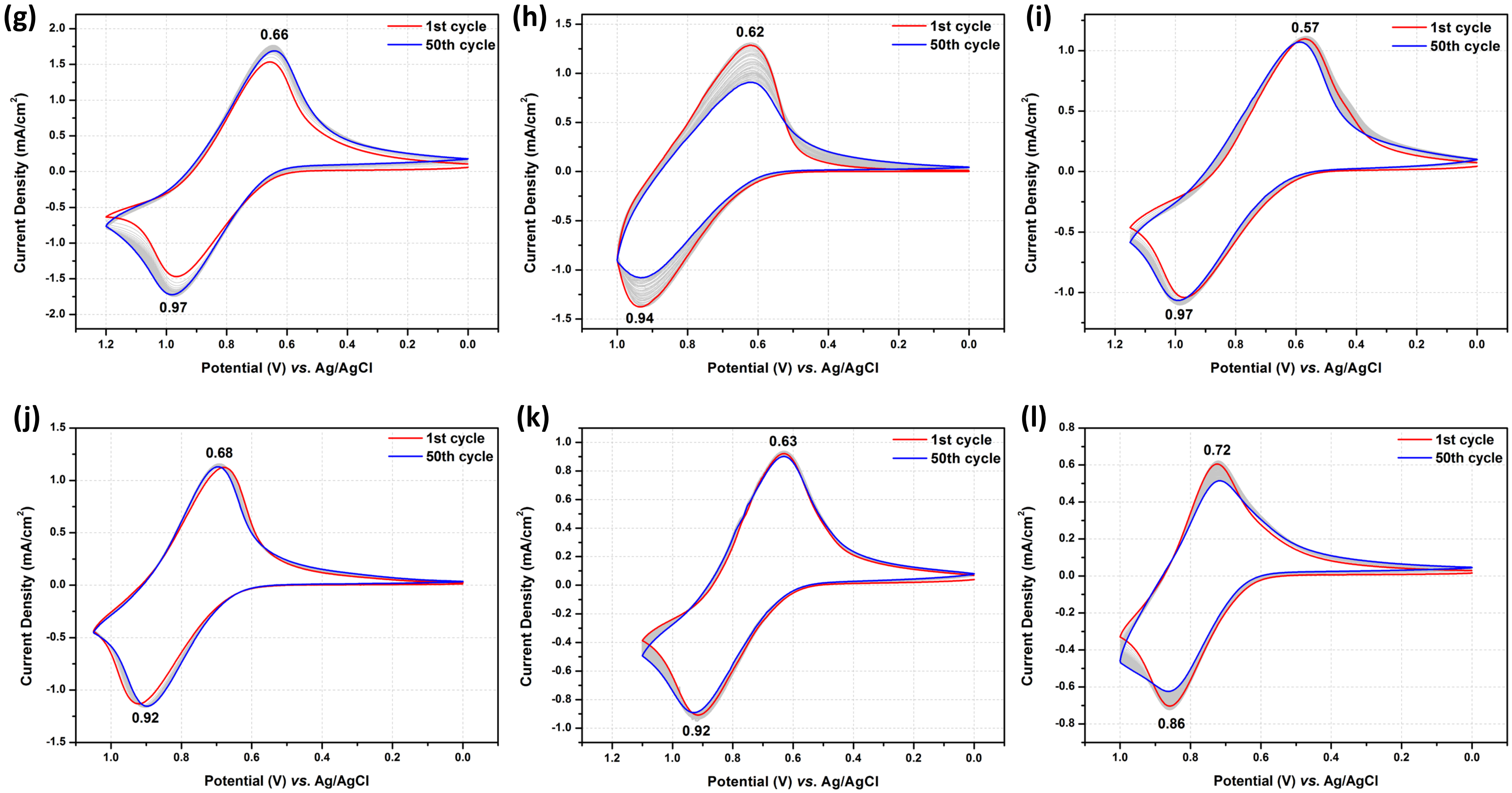

Figure S16. Repetitive CV scans of the cast films of PAIs (a) ***t*-Bu-6a**, (b) ***t*-Bu-6b**, (c) ***t*-Bu-6c,** (d) ***t*-Bu-6d**, (e) ***t*-Bu-6e**, and (f) ***t*-Bu-6f** on an ITO-coated glass substrate in 0.1 M Bu_4NClO_4/MeCN solutions at a scan rate of 50 mV/s.

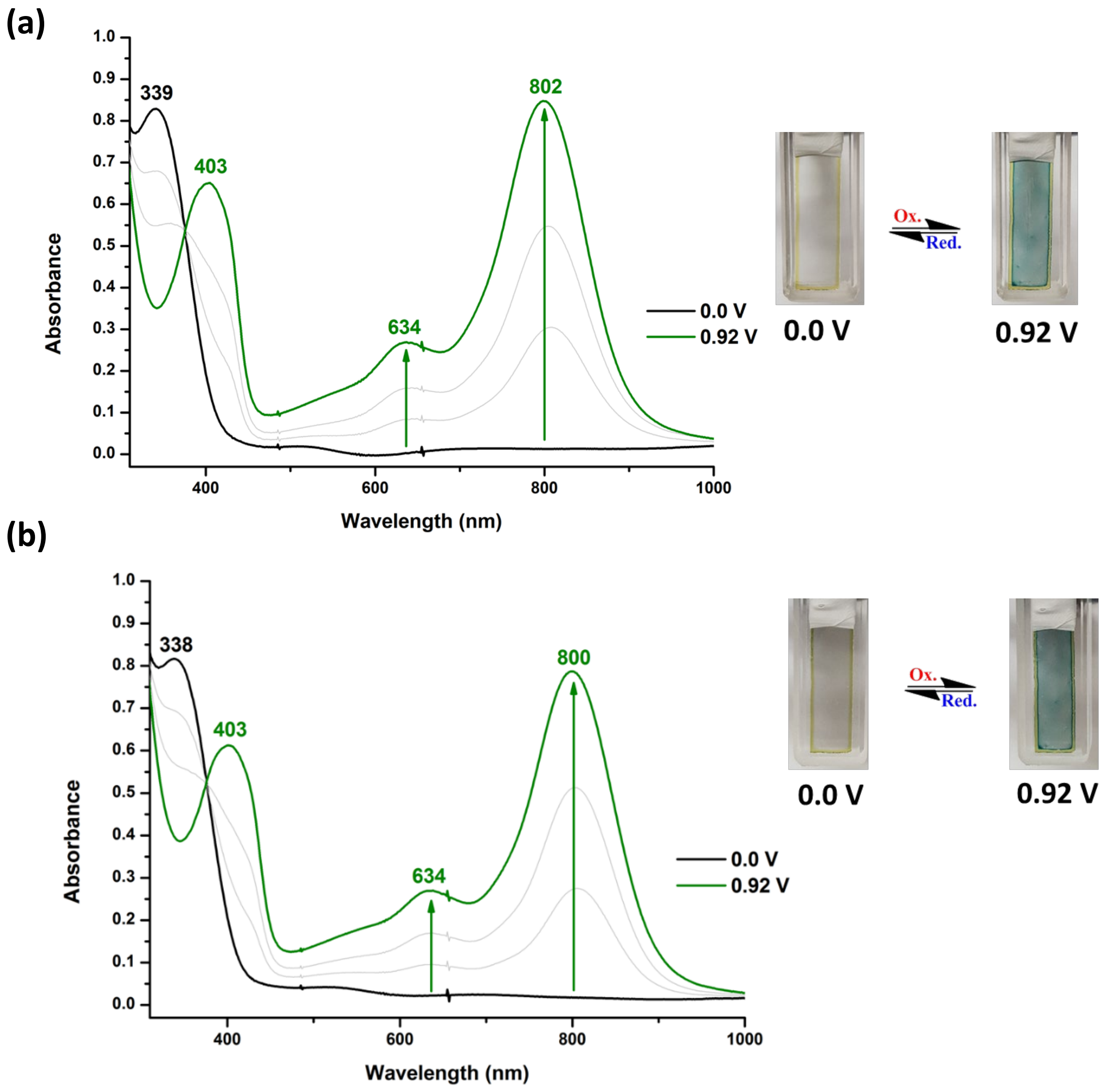

Figure S17. Spectroelectrograms and color changes of PAI ***t*-Bu-6d** on an ITO-glass slide in 0.1 M Bu_4NClO_4/MeCN at various applied voltages (a) first cycle and (b) 50th cycle.

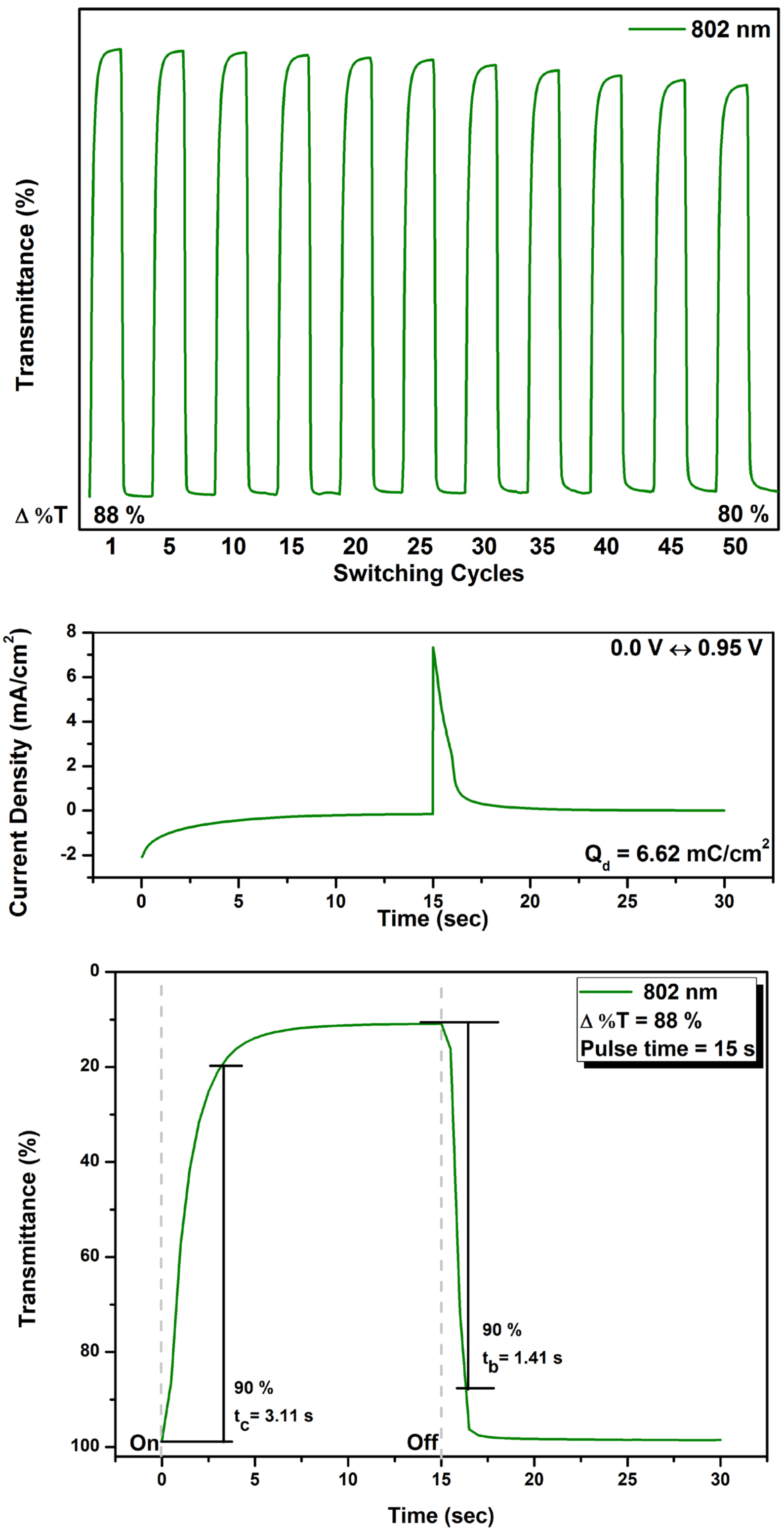

Figure S18. Potential step absorptiometry of the cast film PAI ***t*-Bu-6d** of on ITO-glass slide (in MeCN with 0.1 M Bu_4NClO_4 as a supporting electrolyte) by applying a potential step between 0 and 0.95 V for a resident time of 15s at 802 nm.
